# Controlled Fabrication of Bioactive Microtubes for Screening Anti-Tongue Squamous Cell Migration Drugs

**DOI:** 10.3389/fchem.2022.771027

**Published:** 2022-01-21

**Authors:** Rongbing Tang, Lu Yang, Liheng Shen, Xuan Ma, Yinfeng Gao, Yuan Liu, Zhen Bai, Xuemei Wang

**Affiliations:** School of Stomatology, Lanzhou University, Lanzhou, China

**Keywords:** 3D culture, alginate hydrogel, NSAIDs, human tongue squamous cell carcinoma cells, migration

## Abstract

The treatment of tongue squamous cell carcinoma (TSCC) faces challenges because TSCC has an aggressive biological behavior and manifests usually as widespread metastatic disease. Therefore, it is particularly important to screen out and develop drugs that inhibit tumor invasion and metastasis. Two-dimensional (2D) cell culture has been used as *in vitro* models to study cellular biological behavior, but growing evidence now shows that the 2D systems can result in cell bioactivities that deviate appreciably the *in vivo* response. It is urgent to develop a novel 3D cell migration model *in vitro* to simulate the tumor microenvironment as much as possible and screen out effective anti-migration drugs. Sodium alginate, has a widely used cell encapsulation material, as significant advantages. We have designed a microfluidic device to fabricate a hollow alginate hydrogel microtube model. Based on the difference in liquid flow rate, TSCC cells (Cal27) were able to be evenly distributed in the hollow microtubes, which was confirmed though fluorescence microscope and laser scanning confocal microscope (LSCM). Our microfluidic device was cheap, and commercially available and could be assembled in a modular way, which are composed of a coaxial needle, silicone hose, and syringes. It was proved that the cells grow well in artificial microtubes with extracellular matrix (ECM) proteins by LSCM and flow cytometry. Periodic motility conferred a different motor state to the cells in the microtubes, more closely resembling the environment *in vivo*. The quantitative analysis of tumor cell migration could be achieved simply by determining the position of the cell in the microtube cross-section. We verified the anti-migration effects of three NSAIDs drugs (aspirin, indomethacin, and nimesulide) with artificial microtubes, obtaining the same results as conventional migration experiments. The results showed that among the three NSAIDs, nimesulide showed great anti-migration potential against TSCC cells. Our method holds great potential for application in the more efficient screening of anti-migration tumor drugs.

## 1 Introduction

Head and neck cancer (HNC) is the sixth most common malignant tumor in the world, with about 600, 000 new cases each year (Torre et al., 2015; Shield et al., 2017). Tongue squamous cell carcinoma (TSCC) is one of the most common types of HNC with a high morbidity and mortality rate (Siegel et al., 2013; Yu et al., 2021). Due to the abundance of blood vessels and nerves around the tongue, as well as its flexibility of movement, TSCC is susceptible to early spread and lymph node metastases, even distant metastases (Stuelten et al., 2018). The 5-years survival rate of advanced TSCC is approximately 50% (Fan et al., 2018). In addition, there are currently no effective drugs available to treat TSCC *in situ* and inhibit the distant metastases in the early stage (Jia B. et al., 2019). Therefore, it is urgent to experimentally screen out drugs that effectively inhibit the invasion and metastasis of TSCC, which could be applied clinically to prevent tumors to spread and improve the survival rate of patients eventually (Fan et al., 2018). Non-steroidal anti-inflammatory drugs (NSAIDs)—including aspirin, indomethacin, piroxicam, nimesulide, and ibuprofen—are a group of medication with diverse structures and similar effects, clinically treating rheumatic diseases and reducing inflammation (Ulrich et al., 2006; Jin, 2021). NSAIDs are widely used for antipyretics, analgesics, platelet aggregation inhibitors, and the prevention of strokes (Zhang et al., 2018). In addition, other positive effects of NSAIDs have been found. Clinical and epidemiological studies have revealed that frequent use of aspirin and other NSAIDs could reduce the risk of several cancers (colon cancer, stomach cancer, breast cancer, and lung cancer) (Gilligan et al., 2019; Ekanem et al., 2020; Hsieh et al., 2020; Zhang et al., 2020), and improve survival rate after diagnosis. It has been confirmed that aspirin could reduce migration and invasion of TSCC *via* the PI3K-Akt and focal adhesion pathways (Zhang et al., 2018). There is a wide variety of NSAIDs, and whether all of them have anti-tumor migration has not been studied yet.

Traditional cell cultures *in vitro* are two-dimensional (2D) and are used to observe various biological behaviors of cells and mechanisms of cancer progression, as well as the drug discovery process. (Yamada and Sixt, 2019; Dragoj et al., 2021). However, 2D cell culture models *in vitro* fail to correctly imitate the three-dimensional (3D) environment *in vivo*. When cultured in 2D environment, cells often exhibit abnormal behaviors: abnormal morphology, changes in response to the drug, and loss of differentiation phenotype (Duval et al., 2017). Migration behavior during tumor progression is the result of cell polarization, mechanical coupling, and the establishment and maintenance of cytoskeletal dynamics. There is multi-scale regulation in the complex internal environment (Grinnell, 2003). In addition, cells *in vivo* may not only receive signals on one side but also receive signals from all directions (Baker and Chen, 2012). Therefore, cell migration is considered to be different in dimensionality (Rebelo et al., 2018; Young et al., 2018; Monferrer et al., 2020). The scratch assay is typically used to measure basic cell migration parameters such as speed, persistence and polarity. Cells are grown to a confluent state and a thin “wound” is scratched with the tip of a pipette. Cells at the edge of the wound will migrate into the wound space. However, the scratch assay is 2D and different from the movement of tumor cells *in vivo*. The mechanical movement of the tongue is flexible and frequent, which further promotes the migration of TSCC cells. Therefore, it is also necessary to consider the different states of motion between the cell culture environment and the tissue environment, and the influence of tongue muscle movement on cell migration needs to be taken into account as much as possible.

It is time to improve traditional cell culture to better simulate the complex environment *in vivo* (Pampaloni et al., 2007; Duval et al., 2017). The 3D cell culture models have emerged for studying cancer cell invasion so far, which better imitates the internal environment (Chung et al., 2009; Engel et al., 2015; Wagner et al., 2019; Chang et al., 2020). Due to the variability of morphology and the excellent biocompatibility, hydrogels are widely used in many kinds of research about cell migration in 3D environments (Sugimoto et al., 2018). Currently available methods of hydrogel formation include covalent bonding, photopolymerization, thermo-gelation hydrogels, cryo-gelation and so on. Among the present strategies for preparing hydrogels suitable for cell encapsulation, chemical crosslinking offers immense superiority over other approaches, which can operate under mild reaction conditions with minimal invasiveness for local delivery (Huang et al., 2017). Microfluidics, involving of exquisite manipulation of liquids of small volumes within micrometer-size channels, has been emerging as a promising method for preparing chemically crosslinked hydrogel with well-defined characteristics. Owing to its superior capability in the manipulation of the flows of multiple fluids, microfluidics has offered a powerful technology for rational design and fabrication of hydrogel materials with complicated and precisely tunable sizes, shapes, structures and compositions (Zhao et al., 2020).

Alginate is a polymer material that is composed of β-D mannuronic acid M units and α-l-guluronic acid G units (Martinsen et al., 1989), assembled into a block copolymer (Augst et al., 2006), and gelated of anionic alginate polymers induced by divalent cations. Due to the lack of extracellular matrix (ECM), hydrogels can only simply encapsulate cells and cannot adhere extensively (Caliari and Burdick, 2016), which could not be the same as the microenvironment with cell-to-cell connections and 3D cell interactions *in vivo* (Onoe et al., 2013). To fully simulate the 3D microenvironment *in vivo*, it is necessary to further search for natural extracellular matrix components. Fibrin is a natural polymer formed during wound coagulation (Weisel and Litvinov, 2013). The selective cleavage of the biglycan protein fibrinogen by the serine protease (thrombin), leads to the formation of fibrin hydrogel colt interacting through a series of disulfide bonds (Brown and Barker, 2014; Deller et al., 2019). However, natural fibrin hydrogels tend to take a long time to form a gel (a few minutes even hours) (Onoe et al., 2013). We expect to shorten the gel formation time to ensure the bioactivity of the fibrin hydrogels and optimize the hydrogel 3D cell culture model, further stimulating the movement of tumor cells *in vitro*.

**FIGURE 1:**
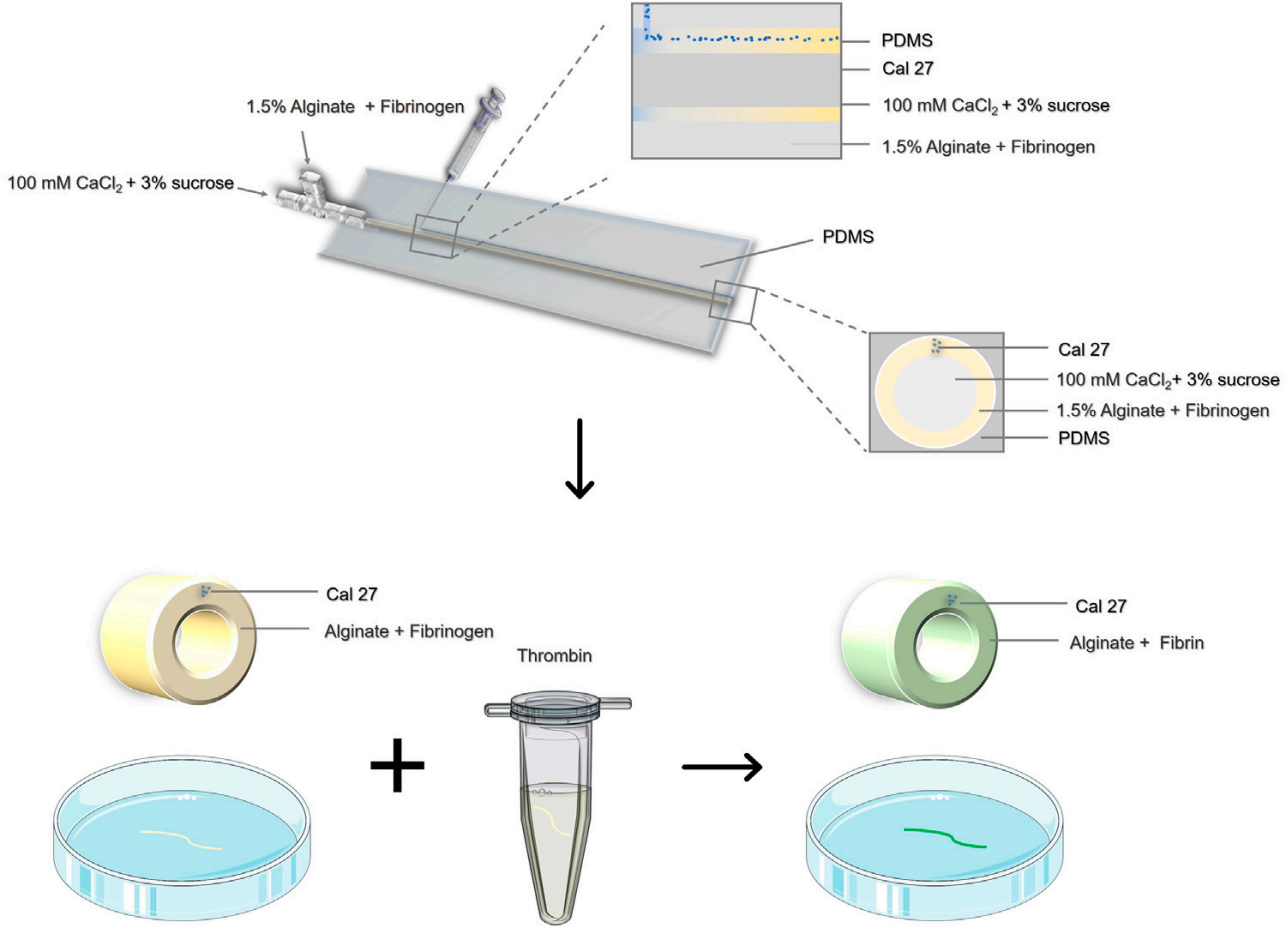
Schematic illustration of the preparation of cell-containing hydrogel microtubes by coaxial microinjection device.

Here we provided a coaxial microfluidic device for preparing hydrogel microtubes (Jia L. et al., 2019; Wang et al., 2019), and introduced the pre-gel solution containing Cal27 cells through a longitudinal ultra-fine needle (Figure 1). The relative flow rate of the hydrogel precursor liquid was controlled to achieve special 3D cell culture structures. We explored the influence of liquid flow rate on the morphology of microtubes and cell arrangement, which could be easily varied to achieve robust fabrication of hollow microtubes with tunable dimensions. To improve cell adhesion and cell viability, we chose alginate-fibrin composite hydrogel to prepare cell-laden hollow microtubes. It has been proved that the cells grew in good condition though cell live/dead staining and flow cytometry. The microtubes had sufficient mechanical strength and could be used to simulate the motion state *in vivo*. It was encouraging that tumor cells maintained physiological activity for 3 days. Through the observation of cell-containing hydrogel microtubes, we identified that three common NSAIDs had anti-tumor cell migration potential, which could be applied the early treatment of TSCC clinically in the future.

## 2 Materials and Methods

### 2.1 Materials

We obtained calcium chloride (CaCl_2_), sodium chloride (NaCl), and alginic acid sodium salt powder from Sigma-Aldrich (Tokyo, Japan). Sucrose was purchased from China National Pharmaceutical Group Co., Ltd. (Sinopharm, Beijing, China). Fibrinogen and thrombin were purchased from Beijing Solarbio Science and Technology Co., Ltd. (Beijing, China). All chemicals were used without further purification. Three types of non-steroidal anti-inflammatory drugs (aspirin, indomethacin, and nimesulide) were purchased from Sigma-Aldrich (Tokyo, Japan).

### 2.2 Cell Culture Condition

Human tongue squamous cell carcinoma cells (Cal27, ATCC CRL-2095) were stored in the Cell Conservation Center of School of Stomatology, Lanzhou University. After recovery from liquid nitrogen, cells were incubated in a sterile incubator at 37°C, in a water-saturated 5% CO_2_ environment. Cal27 cells were maintained in DMEM (Hyclone, Logan, UT, United States), supplemented with 10% fetal bovine serum (Excell bio, Shanghai, China) and 1% penicillin/streptomycin (Solarbio, Beijing, China).

### 2.3 Coaxial Laminar Flow Microfluidic Device

The inner and outer diameters of the coaxial needle were 17 G and 22 G respectively. All inlets and outlets were connected to gastight syringes with silicone hose and Luer fitting (inner diameter: 1.5 mm). The dual-channel micro syringe pump (LSP02-1B, Longer, Baoding, China) was connected to syringes to precisely control the flow rate of the hydrogel precursor fluid. Another single-channel microinjection syringe pump was used to control the precise injection of the cell suspension. The pumps were purchased from Baoding Longerpump Co., Ltd. (Baoding, China).

### 2.4 Manufacture of PDMS Fixture

The PDMS-cured mold and fixed parts were designed by Autodesk Auto CAD 2014 and printed by 3D Printer (ENDER-5S, Creative 3D, Shenzhen, China). When printing, the temperature of the nozzle was 200°C, and the temperature of the hotbed was 60°C. The nozzle spat out polylactic acid (PLA) at a rate of 100 mm/s.

The PDMS curing agent and the precursor were mixed evenly in a volume ratio of 1:10, and the bubbles were discharged by centrifugation (4,500 g, 5 min). The mixed liquid was poured into our printed mold and solidified at room temperature. A hard plastic tube with a diameter of 1.5 mm was inserted in advance into the main mold. Other fixing parts were designed to hold the PDMS, vertical 30G needle and coaxial needle stably.

### 2.5 Formation of Ca-Alginate Hydrogel Microtubes With ECM Protein Containing Cells

For the formation of the hydrogel microtube, we prepared four solutions: 1) a pre-gel cell-containing solution of ECM protein (fibrinogen), 2) a pre-gel solution of alginate containing ECM protein (fibrinogen), 3) a CaCl_2_ solution containing 3% sucrose for gelling, and 4) a thrombin solution (4U), dissolved with PBS, and maintained at 37 °C. Fibrinogen derived from bovine plasma (F8050, Solarbio) was dissolved at 50 mg/ml in PBS buffered saline, and then was adjusted to 5 mg/ml by diluting with PBS. For the shell solution, we used 1.5% Na-alginate solution (V900058, Sigma); 3% w/w Na-alginate was sterilized with an autoclave and mixed with sterilized 2X saline (290 mM NaCl solution) at 1:1 ratio to obtain 1.5% Na-alginate solution in saline (145 mM NaCl solution). Note that in the case of fabricating fibrin microtubes, the 3% w/w Na-alginate solution was mixed with 5 mg/ml fibrinogen solution at a 1:1 ratio. Cells were suspended in the prepared mixing solution at 1.0×10^6^ ml^−1^. The core solution contained 100 mM CaCl_2_ and 3% sucrose (w/w) was sterilized with a 0.22 µm filter.

Before loading solutions into the microfluidic device, the injection device and all tubes were filled with 75% ethanol for 1 h of sterilization. The PDMS was sterilized by UV irradiation for more than 1 h. After that, the device was operated as follows: 1) Fill the device with core and shell solution, introducing saline in the channel of the inner instead of the 100 mM CaCl_2_ solution to avoid the blockage at the point of merging of the shell and sheath streams. 2) Connect the injection device, and insert the 30 G injection needle vertically, securing it with PDMS and fixed module. 3) Start the syringe pumps to inject the core, shell, and cell solutions to generate coaxial laminar flow in the device. A pre-gel mixing solution containing 1.0×10^6^ tumor cells was injected simultaneously. The flow rates of each core, shell, and cell were Qcore = 500 μL/min, Qshell = 500 μL/min, and Qcell = 350 μL/min, respectively. 4) After forming the desired length of the tube, switch the CaCl_2_ flow to the saline stream again and stop the pumps. The diameter of the microtube can be adjusted by changing the flow rate of the stream. 5) Transfer the microtubes containing cells into 4 ml thrombin solution, incubate at 37°C for 15 min, then transfer to a 6-well plate and add 2 ml complete medium (the liquid level was higher than the hydrogel microtubes). Meanwhile, the cells were observed under the microscope and incubated at 37°C, in a water-saturated 5% CO_2_ environment.

### 2.6 Morphology Characterization of Hollow Microtube

The surface of hollow microtubes was observed by ordinary optics microscope and SEM. Different sizes of hydrogel microtubes could be prepared by varying the flow rate of the core and shell solution, achieving controllability of preparation.

The hollow hydrogel microtubes were suspended in water and observed under the microscope (Zeiss VERT1, United States). Then the prepared hollow microtubes were washed third in water to remove calcium chloride, and the samples were dehydrated in a freeze dryer. Glue the sample to the conductive adhesive on the sample stage, and after spraying gold on the particle sputtering instrument for 30 s, the surface and cross-section of the sample were observed by SEM (Thermo Fisher Scientific, Apreo S, United States). Microspheres with green fluorescence were added into the sodium alginate solution, and the cross-section of the hollow microtubes was viewed through LSCM (Olympus Fluoview FV1200, Tokyo, Japan).

### 2.7 Cell Characterizations

After CFSE dye staining, Cal27 cells were observed for their behavior by an inverted microscope (Zeiss VERT1, United States) and LSCM (Olympus Fluoview FV1200, Tokyo, Japan). Cells encapsulated in microtubes were stained with Calcein-AM/PI Cell Live/Dead Assay Kit (Beyotime Institute of Biotechnology, Shanghai, China) to investigate the viability of cells.

### 2.8 Apoptosis Flow-Cytometry Assay

The cells microtubes cultured in 6-well plates were slightly removed, and the cells were stained with Annexin V-FITC/PI Cell Live/Dead Assay Kit (Beyotime Institute of Biotechnology, Shanghai, China) under darkness for 20 min at room temperature. Subsequently, cell apoptosis was analyzed by flow cytometry (Biosciences, LSRFortessa, United States).

### 2.9 Cell Viability Assay

The Cal27 cells were plated in 96-well culture plates at a density of 5 × 10^3^ cells per well. After 24 h of incubation, the cells were treated with aspirin, indomethacin, and nimesulide for the indicated periods. The cell viability was measured by the Cell Counting Kit-8 (CCK8, solarbio, Beijing, China) assays.

### 2.10 Scratch Assay

The scratch assay was classic to analyze the cell migration *in vitro*. The Cal27 cells were seeded in 6-well plates at a density of 1 × 10^6^; after 24 h, a perpendicular scratch was generated on the surface of the plate using a 1,000 μL pipette tip, followed by extensive washing with PBS to remove cell debris. Then, we incubated the plates with DMEM medium containing 1% FBS and treated cells with aspirin, indomethacin, and nimesulide. The photographic images were taken by the light microscope (Zeiss VERT1, United States) at the indicated time after the drug treatment.

### 2.11 Periodic Movement of Microtubes

We purchased a shaker (SLK-03000-S, United States) to perform the periodic motion of microtubes *in vitro*, and the vibration frequency was 100 Hz. The cell culture plate was put on the shaker for 1 h every 4 h to keep the microtubes in motion.

### 2.12 Cross-Sectional Observation of Cell Microtubes

TSCC cells labeled with CFSE dye were distributed in the wall of microtubes in an orderly manner. To observe cell migration behavior, it is necessary to take cross-sectional images of microtubes. First, the cell microtubes were immersed in 0.2% sucrose solution for 1.5 h. Then the microtubes were transferred to a 1:1 mixture of sucrose and O.C.T. to immerse for 2 h. The microtubes were then transferred to O.C.T. solution. After 4 h, fresh O.C.T. solution was replaced and the immersion of microtubes was continued for 6 h. Next, the microtubes were placed parallel to each other and frozen at −80°C for 30 min. Then microtubes were sliced into 20 μm sections and by freezing microtome and observed through the LSCM (Olympus Fluoview FV1200, Tokyo, Japan).

The position of the cell relative to the center of the microtubule was marked based on a zero point using the microtubule center as the coordinate axis. The violin charts showed the cell migration trend. Each experiment was repeated three times, and the results were overlay displayed.

### 2.13 Statistics Analysis

At least three samples were tested for each experiment and all data were reported as mean ± SD. The statistics were analyzed by GraphPad prism software (ver. 8. 0. 2; GraphPad software Inc., United States). Independent unpaired two-tailed Student’s t-tests were used for comparing between two groups. *p* < 0.05 was considered to be significant.

## 3 Results and DISCUSSION

### 3.1 Preparation and Characterization of Cell-Loaded Hydrogel Microtubes

#### 3.1.1 Size-Controllable Hydrogel Microtubes

Alginate is one of the most commonly material for the scaffold of 3D cell culture model due to its outstanding properties, such as excellent biocompatibility, allowing diffusion of gases and nutrients, and stable chemical properties (Namgung et al., 2021). Additionally, alginate is transparent and subsequent experiments can be visualized to ensure stability during injection. (Dragoj et al., 2021). The coaxial microfluidic device was designed and manufactured to precisely fabricate hydrogel microtubes, which was consisted of a coaxial needle and gel channels. The gel channels had an inner diameter of 1.5 mm and were connected to inlets and outlets of the coaxial needle, providing the adequate structure for laminar flows to fabricate calcium alginate microtubes. The 1.5% alginate solution was the outer layer fluid and flowed through the external needle (17 G, diameter:1.5 mm). Through the internal needle (22 G, diameter: 0.7 mm), the 100 mM calcium chloride (CaCl_2_) in 3% w/w sucrose solution was the inner layer fluid (Supplementary Figure S1). When the two fluids came into contact, ionic cross-linking between divalent calcium ions and alginate immediately occurred, and a hollow tubular structure was obtained in the pre-prepared gel channel.

**FIGURE 2:**
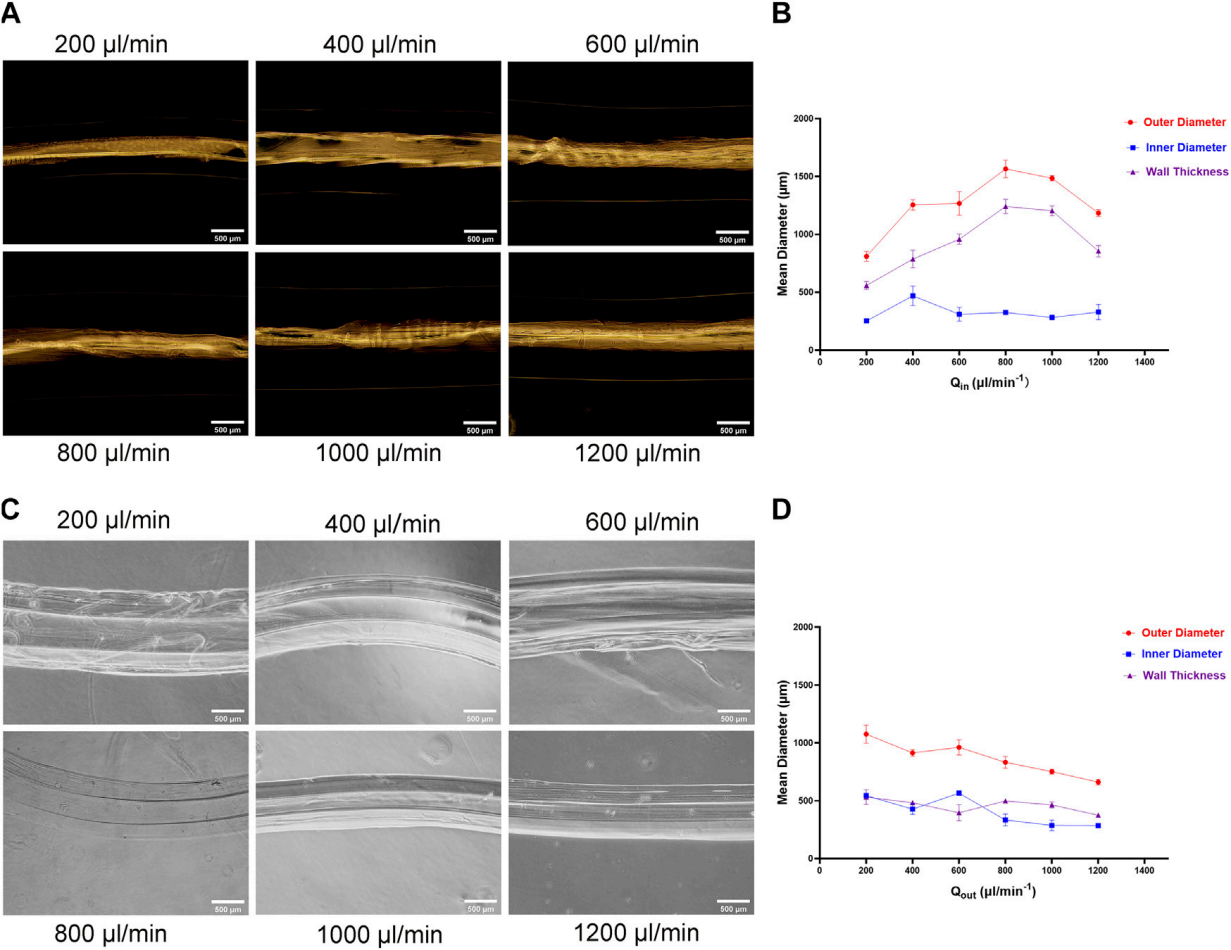
Influence of Outer and Inner Liquid Flow Rate on Microtubes **(A)** The photograph of the prepared hydrogel microtubes: when the flow rate of the outer liquid was consistent with 1,000 μL/min, the flow rate of the inner liquid was 200 μL/min, 400 μL/min, 600 μL/min, 800 μL/min, 1,000 μL/min, 1,200 μL/min, respectively, the scale is 500 μm **(B)** The line graph shows the relationship between the inner liquid flow rate and the microtube structure **(C)** Photograph of the prepared hydrogel microtubes: the inner liquid flow rate was consistent with 400 μL/min, and the outer liquid flow rate was 200 μL/min, 400 μL/min, 600 μL/min, 800 μL/min, 1,000 μL/min, and 1,200 μL/min, respectively, the scale is 500 μm **(D)** The line graph shows the relationship between the outer liquid flow rate and the microtube structure.

Then we examined effects the inner flow (CaCl_2_ solution) and outer flow (alginate solution) rate on the inner diameter, outer diameter, and wall thickness of hydrogel microtubes. When the flow rate of the outer layer (alginate solution) was 1,000 μL/min and the inner increased from 200 μL/min to 1,200 μL/min, the change in the inner diameter is insignificant (Figures 2A,B). The outer diameter and thickness of the microtube wall both increased when the inner fluid rate increased from 200 μL/min to 800 μL/min. Among them, the maximum outer diameter was 1,650.157 μm. While the inner fluid rate exceeded 800 μL/min, the outer diameter and thickness of the microtube wall decreased instead. When the flow rate of the inner layer (CaCl_2_ solution) was 400 μL/min and the outer was from 200 μL/min to 1,200 μL/min, both outer diameter and inner diameter of the microtubes decreased. The outer diameter decreased from 1,156.468 μm to 634.005 μm and the inner was from 560.997 to 264.194 μm. Subsequently, the thickness of the microtube wall decreased by 120 µm on average (Figures 2C,D). Table 1, 2 showed the average range of variation in microtubule structure, revealing more visually the structural variation and high controllability of the microtubes. The results demonstrated the morphology of microtube (e.g. outer, inner diameter, and microtube wall thickness) could be precisely adjusted to satisfy various applications.

**TABLE 1:** Average value of microtubule structure under different inner liquid flow rates.

Qin μL/min	Outer diameter/μm	Inner diameter/μm	Wall thickness/μm
200	809.763 ± 34.412	251.581 ± 11.995	558.183 ± 28.080
400	1,254.784 ± 36.756	468.351 ± 68.838	786.433 ± 62.269
600	1,267.041 ± 83.561	309.1 ± 48.545	957.941 ± 35.735
800	1,565.537 ± 62.567	324.387 ± 15.547	1,241.150 ± 49.876
1,000	1,485.153 ± 18.952	281.927 ± 16.202	1,203.227 ± 34.445
1,200	1,183.366 ± 23.827	328.847 ± 54.030	854.519 ± 40.122

**TABLE 2:** Average value of microtubule structure under different outer liquid flow rates.

Qout μL/min	Outer diameter/μm	Inner diameter/μm	Wall thickness/μm
200	1,076.859 ± 63.616	545.026 ± 11.947	531.830 ± 51.842
400	914.382 ± 24.241	427.732 ± 37.290	483.317 ± 13.827
600	962.742 ± 53.464	565.706 ± 4.079	397.037 ± 57.019
800	832.359 ± 41.597	333.942 ± 42.553	498.418 ± 10.059
1,000	752.008 ± 16.952	287.35 ± 37.471	464.658 ± 21.021
1,200	661.684 ± 20.229	285.735 ± 17.352	375.949 ± 14.110

**FIGURE 3:**
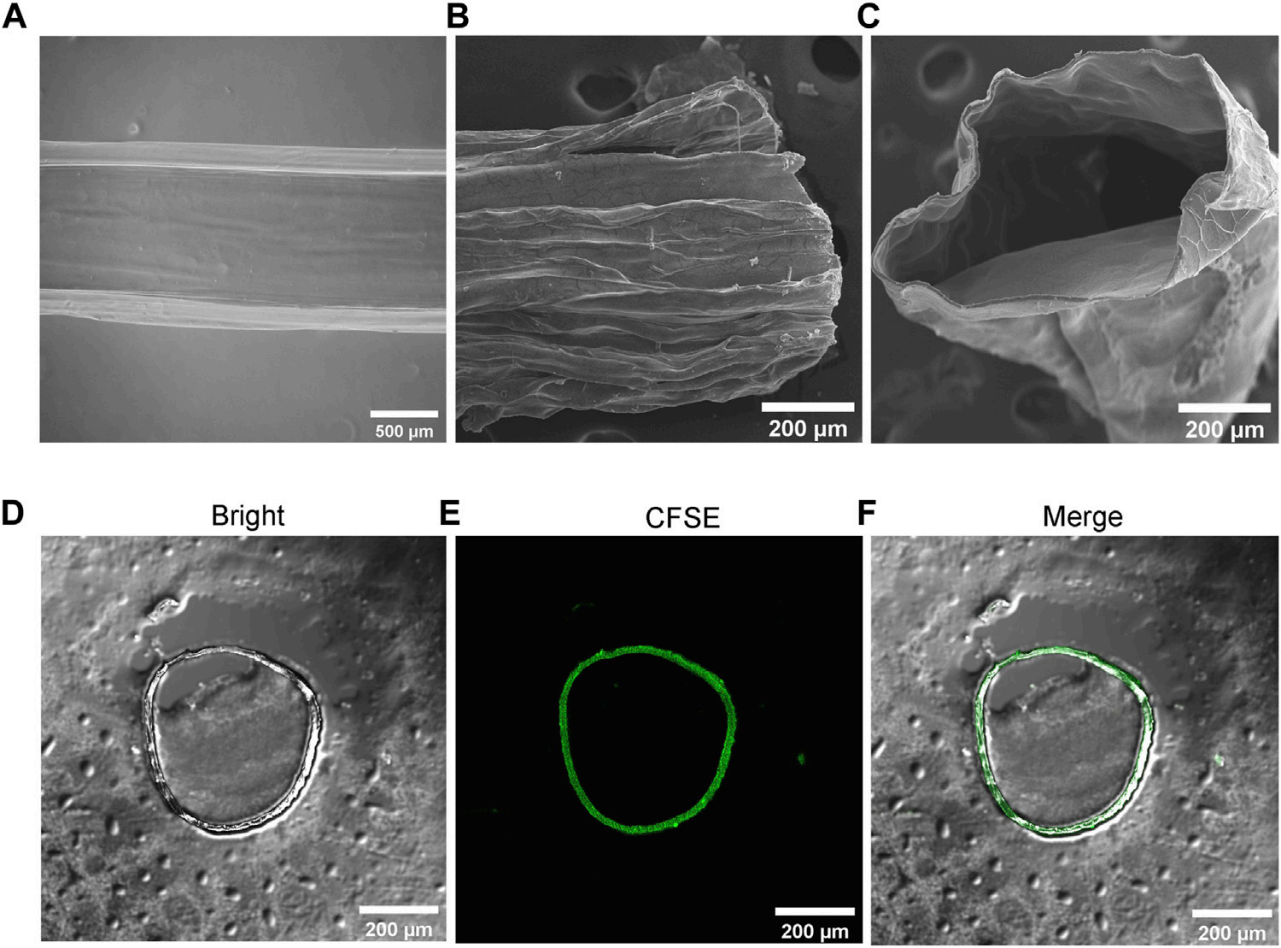
Structure Characterization of Hydrogel Microtubes **(A)** The microscopic image of microtube, the scale is 500 μm; SEM image of **(B)** longitudinal section and **(C)** cross-section of the microtubule, the scale is 200 μm **(D–F)** The cross-section of hollow microtubes was observed with CLSM, after adding microspheres with green fluorescence to the microtubes, the scale is 200 μm.

In addition, we found that the relative flow rates of the two liquids were different, which particularly affected the uniformity and stability of the microtubule structure in particular (Supplementary Figure S2). We chose the same flow rate (500 μL/min) to ensure accurate microtube morphology, uniformity and sufficient wall thickness for cell encapsulation, proliferation, and migration. The generated transparent microtubules were observed by microscopy (Figure 3A). The SEM results confirmed that microtubes possessed a typically tubular (Figures 3B,C). To further observe the structure, microtubes were cut into sections to observe their cross-sectional structure. The results of LSCM showed that microtubes were successfully labeled with green fluorescent microspheres and the cross-section of the microtubes was the regular loops (Figures 3D–F). We determined the optimal parameters for producing hydrogel microtubes with the best tubular shape and certain wall thickness by using micro-injection pumps and coaxial needles, and other devices.

#### 3.1.2. Preparation and Characterization of Cell Loaded Hydrogel Microtube

There are challenges to accurately evaluate the effect of drugs on cell migration. Firstly, the cells should be completely encapsulated with microtubes and evenly distributed. Secondly, the hydrogel microtubes should provide a suitable environment for cells to live in, such as scaffold support, mechanical strength, nutrient delivery, cell-binding sites, biologically active ingredients, and so on. Thirdly, diverse structures of microtubes carrying cells should be easily constructed by changing parameters for different functions. To address these difficulties, we designed a cell loading channel based on hydrogel microtube, which is perpendicular to the gelation channel. The cell-contained solution was injected into the gelation channel through the cell loading channel by a 30 G needle. When the alginate was in a state of incomplete ionic cross-linking, the outer layer fluid in the gelation channel drove cells to form a linear arrangement in the microtube wall. The ionically cross-linked calcium alginate hydrogel acted as a mechanically stable scaffold that maintains the original structure for loading cells. The molecules and proteins that met cell growth were transported through calcuim alginate microtubes transport in the medium on a diffusion basis. However, alginate usually must be modified with an adhesive ligand, such as RGD, to enable cell attachment (Caliari and Burdick, 2016)**.** We tried utilize natural extracellular matrix (ECM) to endow microtubes with cell-binding sites and other properties of tissue *in vivo*. Fibrin is a natural part of the organism and has considerable biocompatibility, facilitating cell adhesion. Moreover, the composite of hydrogel and fibrinogen can be accomplished by simple physical mixing, saving time and economic cost. Our devices compositions are inexpensive, commercially available, and can be flexibly assembled to satisfy researches of various mechanisms.

**FIGURE 4:**
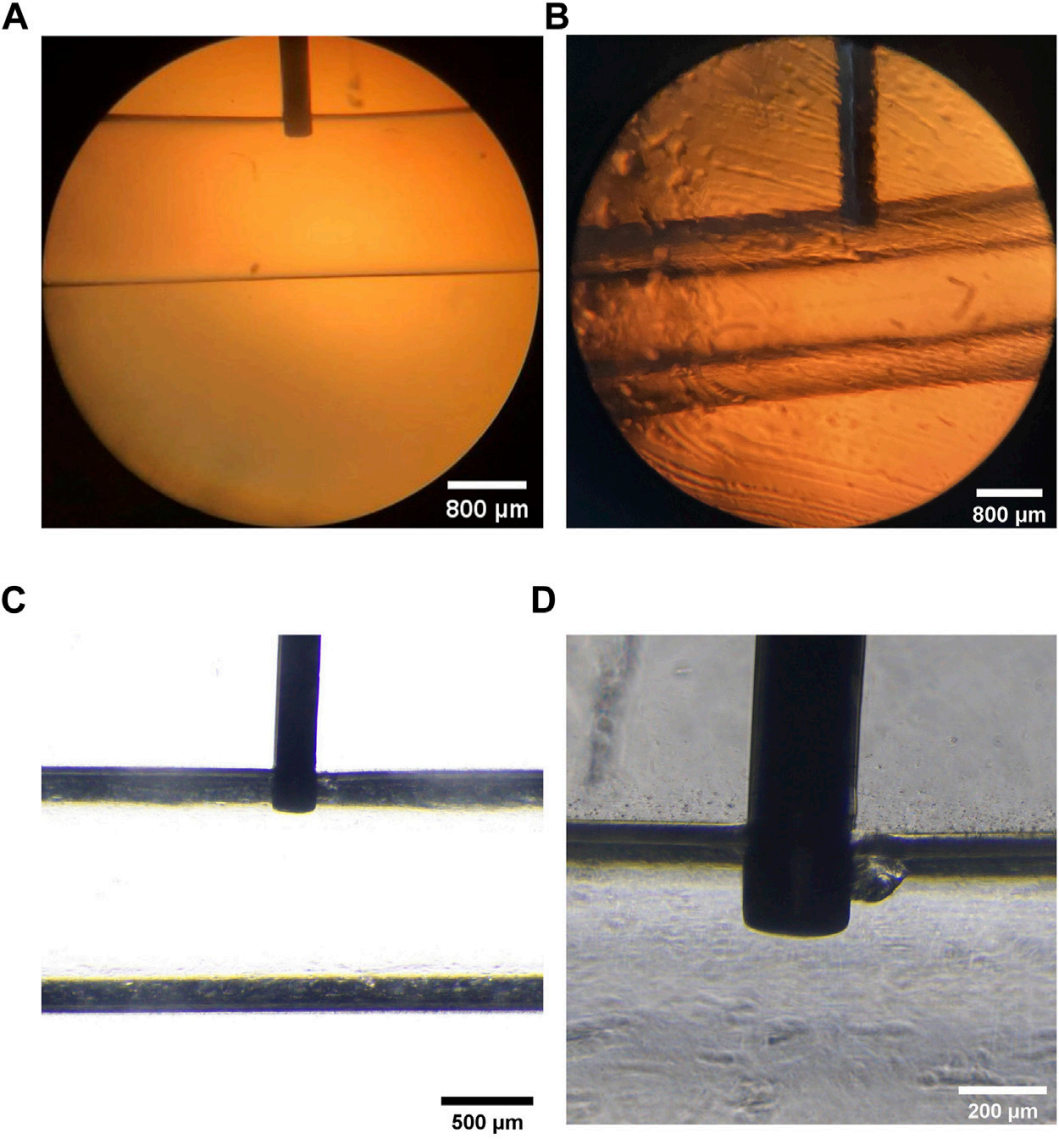
Vertical Injection Depth of the 30 G Needle **(A)** Microscopic image of the PDMS fixed block inserted a 30 G injection needle vertically, the scale is 800 μm **(B)** Microscopic image of internal and external liquid and the vertical injection needle, the scale is 800 μm **(C,D)** The depth of injection perpendicular to the needle was marked under the microscope and measured at 231.803 μm, the scales are 500 and 200 μm, respectively.

Figure 4 showed that the insertion depth of the needle dramatically affected the construction of microtubes. When the insertion depth of needle exceeded the thickness of the microtube wall, the cells would flow out of the channel with the CaCl_2_ liquid. On the contrary, if the depth was not sufficient, cells could not adhere to the wall of the microtube (Supplementary Figure S3). When the insertion depth of the needle reached 230 μm, the cell-containing fluid was uniform and continuous and the microtubes remained integral. Therefore, we fabricated a fixation device by 3D printing to ensure the insertion depth of the needle in the following experiment (Supplementary Figure S4). The depth of the vertical cell suspension entered the wall of the hollow calcium alginate hydrogel microtube was 231.803 μm. The preparation process was stable and efficient (Supplementary Figure S5), and the instrument was inexpensive and commercially available.

**FIGURE 5:**
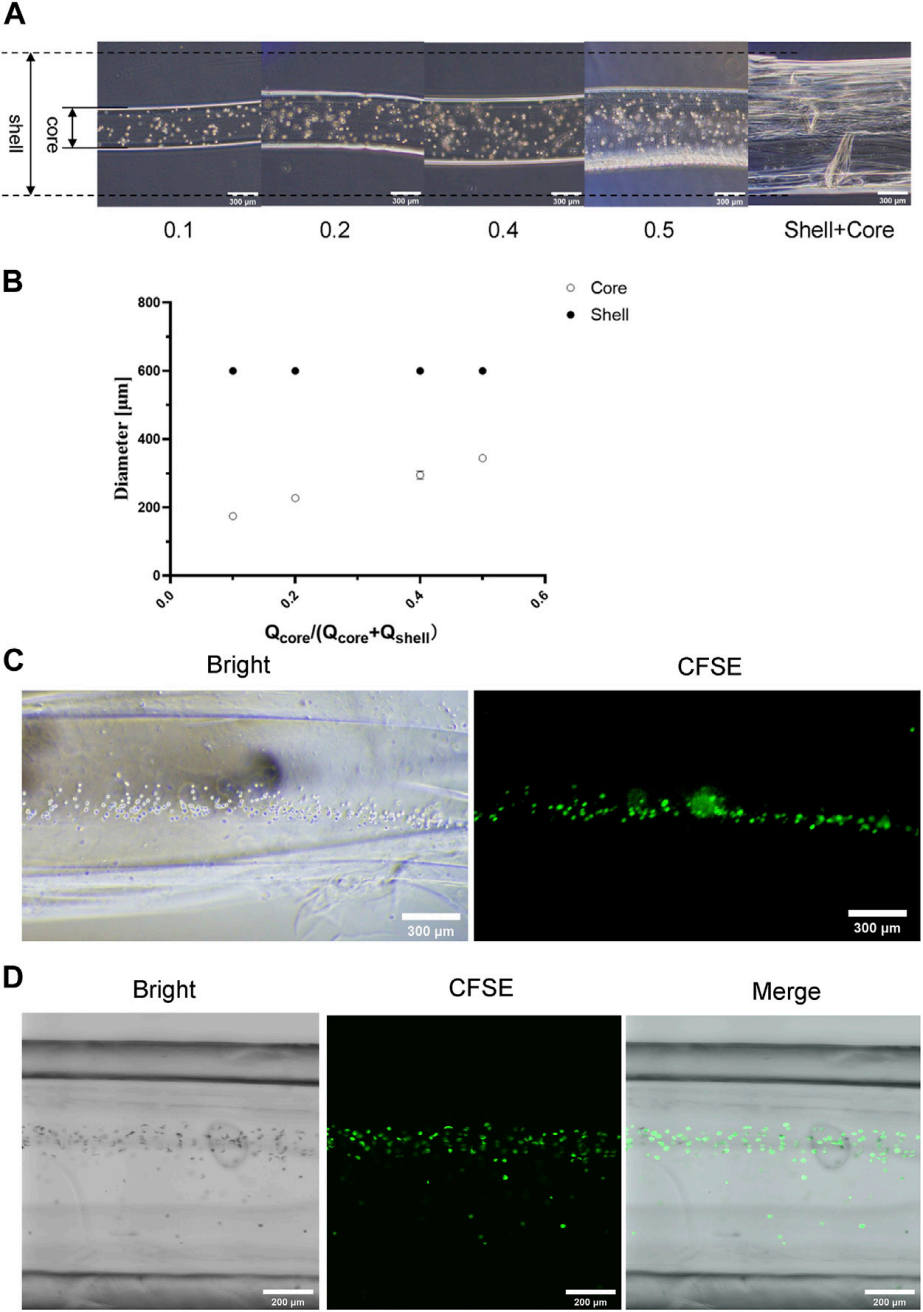
Structure Characterization of Cell Filament **(A)** Microscopic images of formed cell filaments under different flow ratios, the scale is 300 μm **(B)** The relationship between the diameter of the formed cell filament and the flow ratio **(C)** Microscope images of the cell filaments distribution in calcium alginate microtubes, the scale is 300 μm **(D)** CLSM image of cell microfilaments distributed in calcium alginate microtubes, the scale is 200 μm.

We further optimized parameters to obtain suitable cell-loaded microtube structures, such as the flow rates of inner layer fluid, outer layer fluid, and cell-containing fluid. Here we prepared three solutions: 1) the alginate solution containing the ECM and cells is referred to as the core liquid, 2) the alginate solution containing the ECM is the shell liquid, and 3) the CaCl_2_ solution is used for gelling. The sum of the flow rates of the shell and core liquids was 500 μL/min. The ratio of the flow rates of the shell and core liquids was called the relative liquid flow ratio. There was a positive correlation between the diameters of the cell filaments in microtubes and the relative liquid flow ratio, according to the microscope image and the quantitative analysis (Figures 5A,B). With the gradual increasing of the liquid flow ratio, the diameter of the cell filaments showed a steady increase. Table 3 shows the average diameter of cell filaments. As the flow rate ratio increases, the diameter increases from 174.837 to 344.430 μm. We chose the relative liquid flow ratio of 0.4 for subsequent experiments, ensuring that the cell filaments could be distributed evenly on the microtube walls. The flow rate of the cell-containing alginate solution was 350 μL/min. In this way, microtubule morphology was not significantly affected and the cells were arranged in an orderly manner (Figure 5C). The structures of hollow microtubes were observed by CLSM (Figure 5D), and the cells are uniformly distributed in the microtubes. The results demonstrated that the structure of the hydrogel and the arrangement of the cells can be changed by controlling the parameters in the injecting process.

**TABLE 3:** Average diameter of core-shell structure under different core-shell liquid flow ratio.

Qcore/Qcore + Qshell	Core/μm	Shell/μm
0.1	174.837 ± 2.462	592.092 ± 21.128
0.2	227.82 ± 9.958	
0.4	294.813 ± 11.911	
0.5	344.430 ± 7.479	

Alginate hydrogels have great potential to advance 3D cell culture in microtubular structures (e.g. microfibers, microtubes). Now microtubes with alginate core-shell structure have been successfully fabricated with self-designed and fabricated microfluidic devices. Dragoj reported a long-term observation system for three-dimensional glioblastoma based on syringe-extruded alginate fibers (Dragoj et al., 2021). Jorgensen demonstrated the capacity of fabricating hydrogel microtubes of coaxial needle devices (Jorgensen et al., 2021). We have produced hollow hydrogels based on hydrodynamic principles and microfluidic devices. Our equipment allowed flexible control of the size and geometry of the hydrogel microtubes, which have rarely been reported so far. In addition, the devices are reusable, stable and efficient, and can produce hydrogel materials with various cellular arrangements. The microtubes were continuously generated by microfluidic devices, allowing for mass production. A vertical flow containing cells could also be easily and conveniently introduced into the hollow microtubes with our devices.

### 3.2 Influence of Hydrogel Microtubes on Cell Adhesion, Proliferation and Viability

The biocompatibility of hydrogel microtubes was improved by incorporating fibrin, a natural ECM. The cell adhesion, cell proliferation, cell activity and cell migration were tested to evaluate the behavior of cells encapsulated in hydrogel microtubes.

**FIGURE 6:**
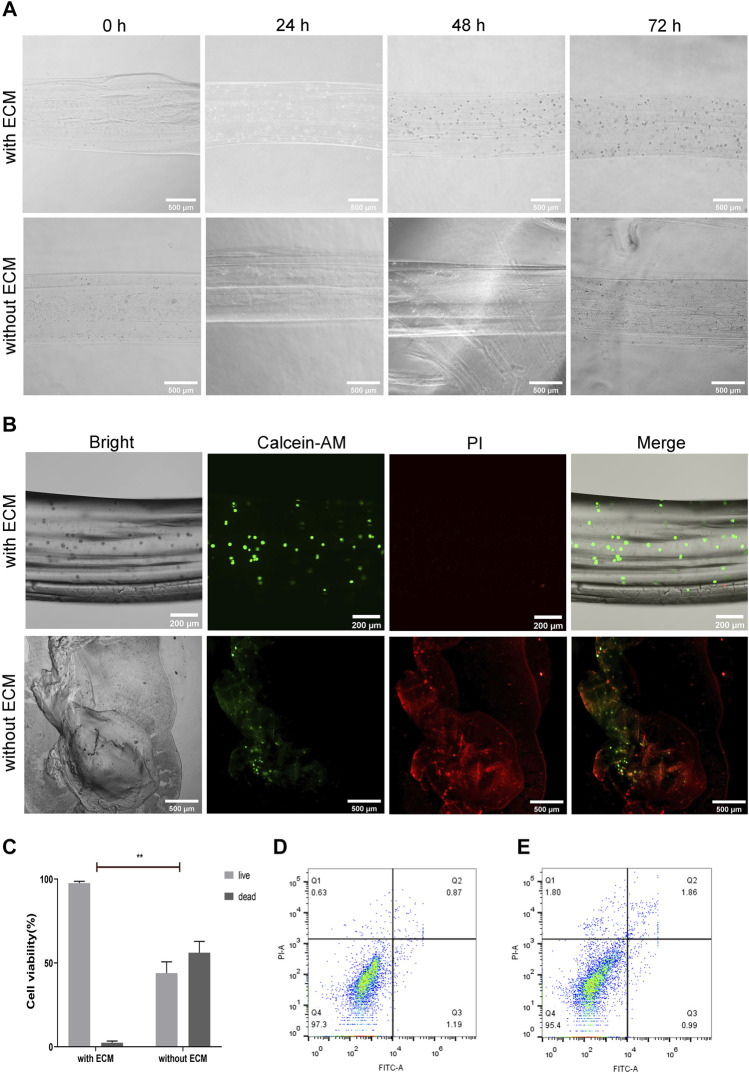
Biocompatibility of Hydrogel Microtubules **(A)** The micrographs of alginate hydrogel microtubes encapsulated with Cal27 cells, with or without ECM, at 0, 24, 48 and 72 h, the scale is 500 μm **(B)** Cal27 cells were encapsulated in alginate hydrogel microtubes with or without ECM and cultured for 72 h. Live cells were labeled with green fluorescence by Calcein-AM and dead cells were labeled with red fluorescence by PI, the scales are 200 and 500 μm, respectively **(C)** Quantitative analysis of live and dead cell staining **(D,E)** Apoptosis was detected by flow cytometry after 48 and 72 h of culture with Annexin V-FITC/PI staining. The apoptosis rate was low in the algnate hydrogel with ECM group.

The Cal27 cells encapsulated in hollow microtubes, with or without natural ECM, were cultured in a complete medium for 24, 48, and 72 h. The cells in the hollow microtubes containing natural ECM exhibited increased proliferation and adhesion (Figure 6A, Supplementary Figure S6). In microtubes with natural ECM, the survival rate of cells was about 97.61%. Moreover, cells were spherical in shape and had direct intercellular contact. The 3D structure of the cell-filled microtubes and the morphology of the cells inside were observed to reveal a clear boundary between the cells and microtubes. On the third day, Cal27 cells maintained spherical and showed a clear spreading. In microtubes without ECM, Cal27 cell proliferation was inhibited, the hydrogel collapsed, and approximately 56.11% of the cells died after 72 h of culture (Figures 6B,C). Some cells leaked out of the microtubes and attached to the wall of the cell culture flask (Supplementary Figure S7).

The results indicated that the natural ECM components could effectively enhance the bioactivity of the hydrogels. The hydrogels containing natural ECM reconstituted the suitable microenvironment that highly mimicked typical cell-cell junctions and 3D cellular interactions of living tissues. The ECM proteins in microtubes regulate cell morphology and physiological functions, and promote cell proliferation. By encapsulating the cells in an ECM-containing hydrogel, the cells could also exhibit intrinsic morphology and function *in vitro*.

In addition, the cell apoptosis in the hydrogel microtubes with ECM was measured by flow cytometry (Figures 6D,E). After 48 h, the percentages of early and late apoptotic cells were 1.19 and 0.87%, respectively. After 72 h, the percentage of early apoptotic cells was 0.99% and the percentage of late apoptotic cells was 1.86%, both of which were less than 2%, indicating that the cells were in good growth condition. The results of flow cytometry indicated that the hydrogel with ECM had no cytotoxic effect on Cal27 cells, but rather promoted cell adhesion and proliferation, consistent with microscopic observations. The design of hydrogel microtubes is based on the advantages of calcium alginate and fibrin, which are biocompatible and support cell growth.

### 3 3 Evaluation of Anti-migration Effects of Drugs

#### 3 3 1. Three Non-steroidal Anti-inflammatory Drugs

The levels of COX-2 and its prostaglandin derivatives, especially prostaglandin E2 (PGE2), were increased in premalignant and malignant lesions of TSCC. The overexpression of COX-2 and increased levels of PGE2 could contribute to the inhibition of apoptosis and promote invasion and metastasis (Zhang et al., 2018). Aspirin irreversibly acetylates cyclooxygenase, thereby inhibiting COX-1 and COX-2. In recent years, the preventive effect of aspirin on tumors has been widely recognized (Drew et al., 2016; Adamička et al., 2021)**.** In addition, aspirin stimulates the production of endogenous anti-inflammatory and pro-resolution mediators, such as aspirin-triggered resolving (AT-RvDs) and lipoxins (AT-LXs). These mediators can counter regulated expression of tumor migration inhibitors (Gilligan et al., 2019).

**FIGURE 7:**
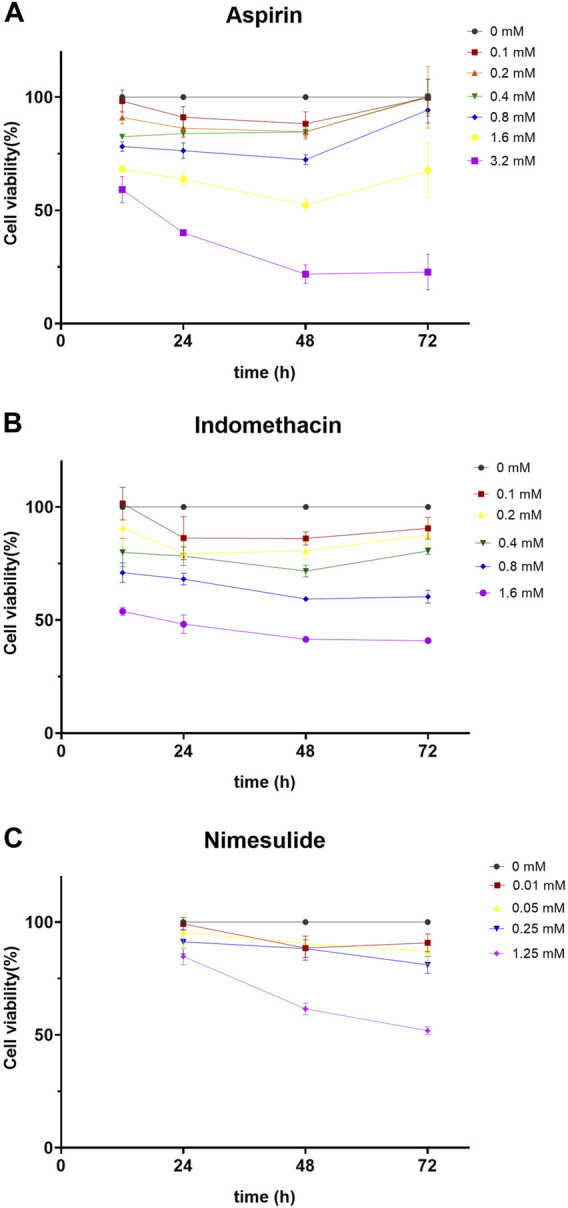
Cell Viability Assay **(A,B,C)** Aspirin, indomethacin, and nimesulide inhibited cell viability in dose- and time-dependent manner. Cal27 cells were treated with different concentrations of drugs and the viability of these cells was assayed with CCK8 for the indicated times.

Other NSAIDs, such as indomethacin and nimesulide, have also been reported to have the ability to inhibit COX-2 (Chen et al., 2021; Xiao et al., 2021). Therefore, we hypothesized that these NSAIDs might prevent the migration of TSCC. The CCK8 assay was used to detect cell viability after treatment with different concentrations of NSAIDs, in order to exclude the effect of drug concentration. Cal27 cells were treated with different concentrations of NSAIDs (aspirin: 0, 0.1, 0.2, 0.4, 0.8, 1.6, 3.2 mM; indomethacin: 0, 0.1, 0.2, 0.4, 0.8, 1.6 mM; nemesulide: 0, 0.01, 0.05, 0.25 mM) and cell viability was assayed at the indicated times (24, 48, 72 h). We found that cell viability was inhibited in a dose- and time-dependent manner. As shown in Figure 7A, the survival rate of Cal27 cells after 72 h was less than 30% when the aspirin concentration was 3.2 mM. At a concentration of 1.6 mM, the survival rate of Cal27 cells for 72 h was about 68%, but at 0.2 mM it was about 99%. Since some concentrations of aspirin affected cell viability, we chose smaller concentrations, 0.1 and 0.2 mM, as representative doses for *in vitro* treatment. Similarly, 0.1 and 0.2 mM were chosen for indomethacin and 0.01, 0.05 and 0.25 mM for nimesulide (Figure 7B,C). These concentrations of NSAIDs had no significant inhibitory effect on tumor cell proliferation and were used for the follow-up experiments.

#### 3 3 2. Two-Dimensional Static Migration Experiment

**FIGURE 8:**
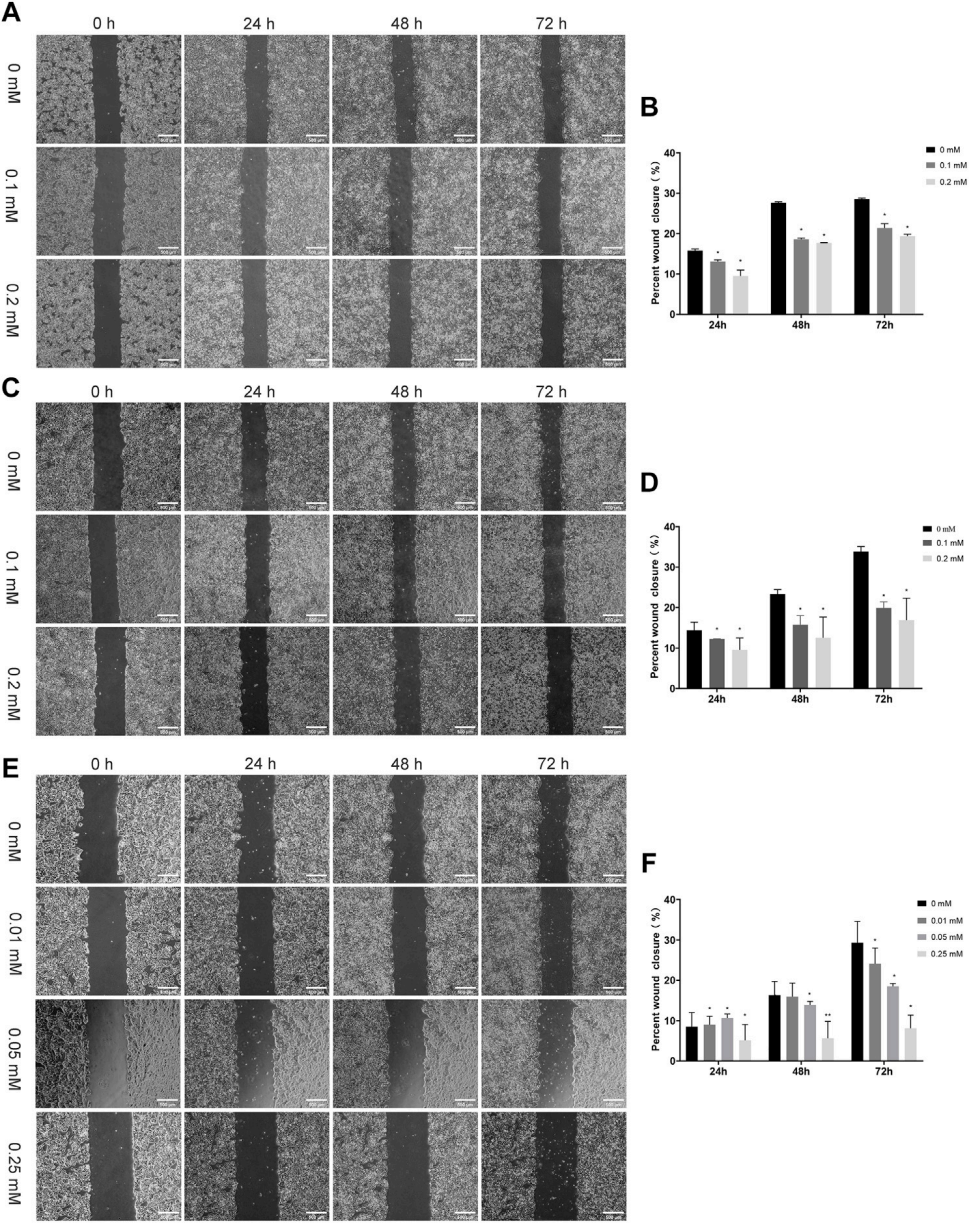
Scratch Assays **(A,B)** Cal27 cells were treated with different concentration aspirin (0.1, 0.2 mM), the scale is 500 μm **(C,D)** Indomethacin (0.1, 0.2 mM), the scale is 500 μm and **(E,F)** Nimesulide (0.01, 0.05, 0.25 mM), the scale is 500 μm. Photographs were taken immediately after the formation of the scratch and when 24 h, 48 and 72 h later. The relative scratch covered area was quantified by ImageJ, *p < 0.05, **p < 0.01. The data were presented as the mean ± SD (n = 3).

We evaluated the effects of NSAIDs (aspirin, indomethacin, nimesulide) on cell migration ability using conventional methods. Scratch assay has long been the most common *in vitro* method for testing compounds with migration-resistant and migration-promoting properties because of its simplicity and low cost (Figure 8). As shown in Figures 8A,B, the scratch-healing rate of the aspirin treatment group decreased significantly compared with the control. Similar results were also observed in indomethacin and nimesulide groups (Figures 8C–F). Scratch assay indicated the anti-migration ability was related to the drug concentration and treatment time. These results suggested that aspirin, indomethacin, and nimesulide had great potential to inhibit the migration of TSCC cells in a 2D culture environment. The biological properties of tumor cells have been largely neglected because the conventional 2D cell culture methods oversimplify the biological environment of tumors.

#### 3 3 3. Three-Dimensional Dynamic Microtubule Model Experiment

**FIGURE 9:**
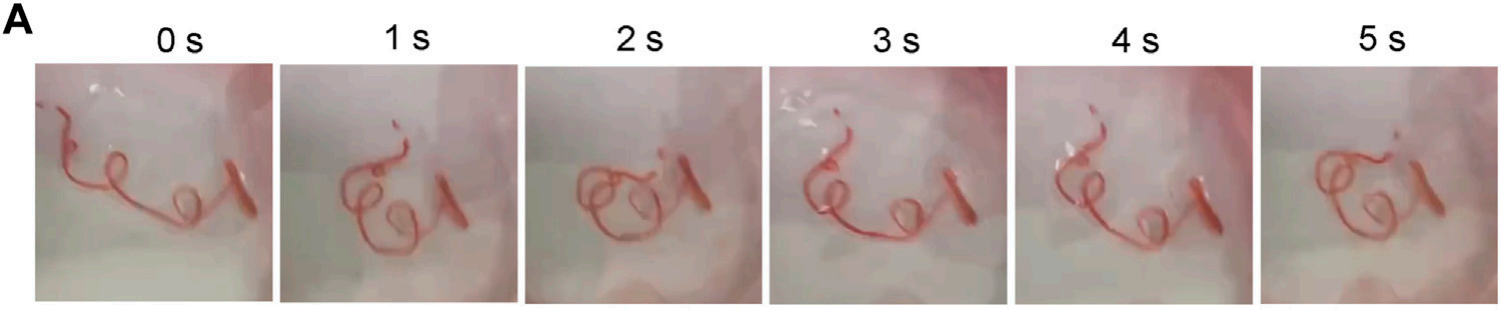
Periodic Movement of Microtubes *in vitro* **(A)** Microtubules perform a periodic movement on a shaker real-time Image of Movement Status within 5 s.

If the cell culture environment is increased from two to three dimensions, it can significantly affect the biological behavior of the cells. Tumor migration in the microenvironment involves many factors, including extracellular matrix and tissue movement characteristics. The extracellular matrix (ECM) is an important component of the cellular environment, regulating cellular behaviors such as cell migration. More importantly, tumor cell motility is dependent on tissue dynamics, so alterations in tissue mechanics can affect cell proliferation and cell migration. While migrating through a mechanically and spatially heterogeneous 3D microenvironment, changes in cell mechanics allow influencing mechanism signaling pathways and cytoskeletal architecture to alter migration and metastasis (Kechagia et al., 2019). Therefore, migration experiments *in vitro* need to further consider the characteristics of tissue dynamics. The periodic motion every 4 h *in vitro* gave the cell microtubes a different dynamic environment (Figure 9). With the sufficient mechanical strength and flexible property of hydrogel, the microtubes could simulate tissue motion by exerting hydrodynamic effects.

**FIGURE 10:**
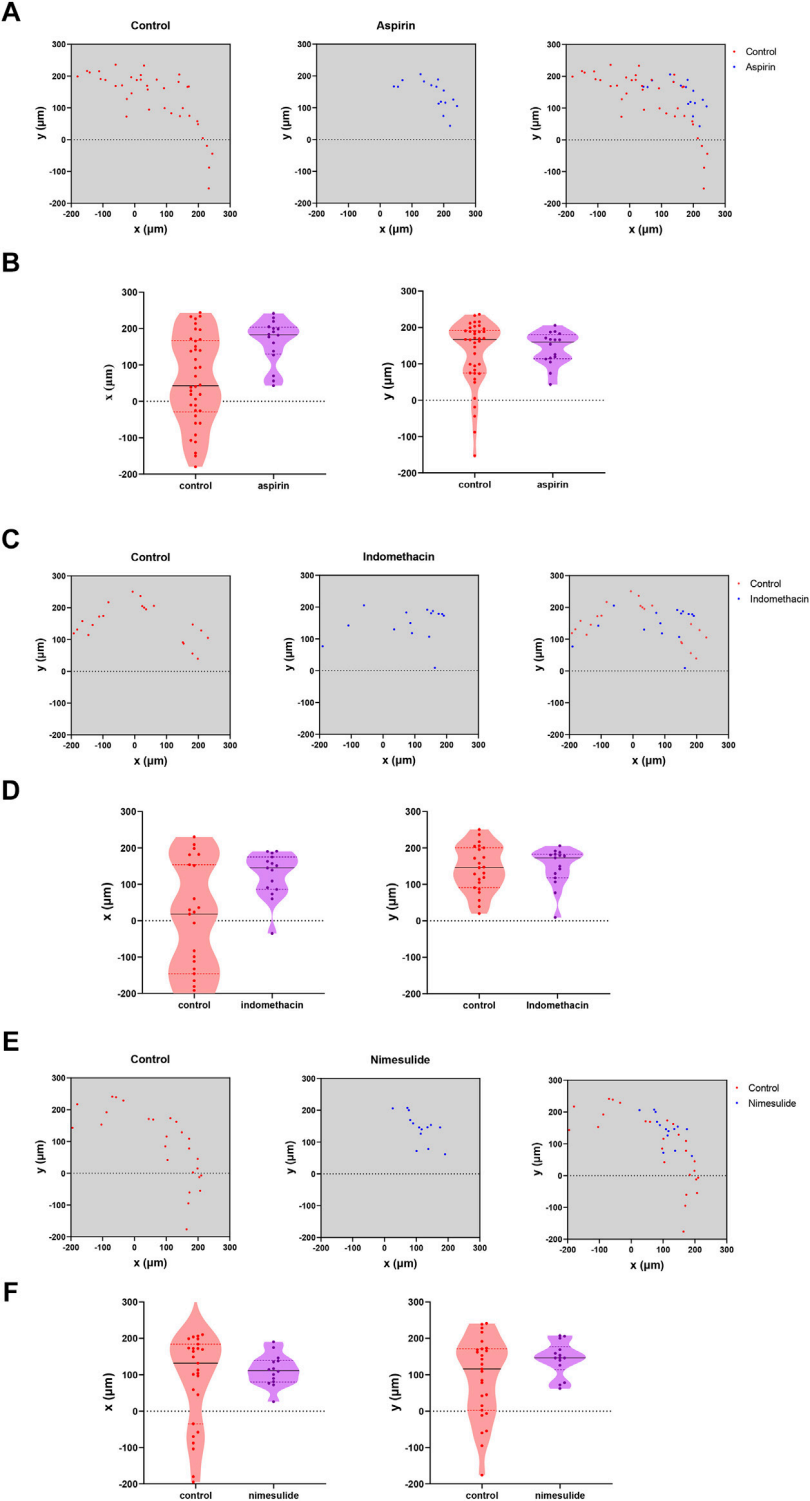
Analysis of NSAIDs’ Anti-Cal27 cells Migration in Microtubes Model **(A)** Quantification of the location of fluorescently labeled cells in microtubes in the aspirin and control groups **(B)** Analysis of the migration trends of cells in the *x*-axis and *y*-axis directions on microtubes in the aspirin- and control groups, respectively **(C, D)** Indomethacin group analysis **(E,F)** Nimesulide group analysis.

We selected the drug concentration with the most significant inhibitory effect for follow-up experiments (aspirin: 0.2 mM; indomethacin: 0.2 mM; nimesulide: 0.25 mM). After 72 h of drug treatment (Figure 10), observation of cell migration on the cross section of microtubes, and the axes of the cells on the figures were used to locate the specific position of cells. The results showed that Cal27 cells in the NSAIDs-treated group had a more concentrated cell arrangement and shorter migration distance than the control group. Quantitative analysis of cell displacement on cross-sections revealed that the migration distances were significantly shorter in the three drug groups compared to the control group. The cell migration distances in the aspirin group ranged from 43.012 to 242 μm in the *x*-axis direction and from 43 to 206.002 μm in the *y*-axis direction. The migration distances in the control group ranged from −180.003 to 244.008 μm and from −236.002 μm to −153.003 μm in the *x*-axis and *y*-axis, respectively. In the indomethacin group, the migration distance in the *x*-axis direction was from −35.014 to 190.011 μm, and the *y*-axis direction was from 9 to 206 μm. And the range of cell migration distances in the nimesulide group was 164.476 and 146.013 μm in the *x* and *y* axis directions, respectively. Compared with the control group of each group, the migration range of cells in the aspirin group was reduced by 225.023 and 226.003 μm in the two directions, indomethacin was 252.335 and 33.689 μm, and nimesulide was 240.624 and 268.96 μm. These differences reflected the inhibitory effect of the drugs in the cell migration, with larger numbers indicating a more pronounced inhibitory effect. Tables 4–6 compared the results of the migration experiment and the cell microtubule model. The 72 h scratch healing area and cell migration range on microtubules show numerical differences between the drug group and the control group. Among three groups, cell dispersion was significantly lower in the nimesulide treatment group, with a smaller range of cell migration distances. The ranges of the *x*-axis and *y*-axis are 164.476 and 146.013 μm, respectively. Nimesulide displayed a superior advantage on anti-migration, implying the great potential for treatment and prevention. In addition, the results of the microtube model were highly reproducible. Our 3D migration model yielded more intuitive experimental results compared to traditional migration experiments. Cell microtubes are compared with the values of the horizontal and vertical migration ranges, which prompts more migration information. Moreover, there are clear advantages of 3D culture compared to 2D culture (Caliari and Burdick, 2016), such as normal cell morphology and cell differentiation phenotype.

**TABLE 4:** The average healing rate of 72 h scratch assay and 72 h cell migration range on microtubes in aspirin groups.

72 h	Scratch assay/%	Cell microtubes/μm	
Control	28.519 ± 0.263	212.006 ± 106.003	194.503 ± 97.251
Aspirin	19.343 ± 0.500	99.494 ± 49.947	81.501 ± 40.751

**TABLE 5:** The average healing rate of 72 h scratch assay and 72 h cell migration range on microtubes in indomethacin groups.

72 h	Scratch assay/%	Cell microtubes/μm	
Control	33.835 ± 1.232	238.68 ± 119.34	115.345 ± 57.612
Indomethacin	16.846 ± 5.455	112.513 ± 56.256	98.5 ± 49.25

**TABLE 6:** The average healing rate of 72 h scratch assay and 72 h cell migration range on microtubes in nimesulide groups.

72 h	Scratch assay/%	Cell microtubes/μm	
Control	29.286 ± 5.281	202.505 ± 101.253	207.487 ± 103.743
Nimesulide	8.101 ± 3.284	82.238 ± 41.119	73.007 ± 36.503

Additional periodic motility provided cells in microtubes with different states of motility, which greatly promoted cell invasion and metastasis (Stuelten et al., 2018). Cell movement is the result of the protrusion of the cell front, adhesion to the extracellular matrix (ECM), and then the adhesion on the cell back accompanied by contraction to squeeze the cell body forward (Blanchoin et al., 2014). The fibrin, a natural ECM, was added to the hydrogel and played a role for cell adhesion. Although the relationship between fibronectin and the dynamic and mechanical properties of cells is unclear, the apparent increase in cell migration after the addition of fibronectin suggested that fibrin promote cell migration behavior.

Moreover, the amounts of cells required in microtube model is far less than the conventional scratch assay. Our microtube model was stable, efficient, time-saving, and reproducible for drug screening. The cells were in the same position in each group of microtubes, allowing for comparison of position changes. Therefore, high-throughput drug screening can be performed to verify the effects of multiple drugs simultaneously and for comparison. In the above research, we successfully screened out three NSAIDs with anti-migration potential in Cal27 cells, among which nimesulide was the most effective, but its mechanism has not yet been elucidated. Further experiments are required before clinical application. It provided a reproducible, well-controlled, 3D cells culture model for future biological studies *in vitro.*

## 4 Conclusions

In summary, we provided a hydrogel-based screening model for anti-migration tumor drugs. Calcium alginate hydrogel microtubes were fabricated using coaxial needles and microfluidic devices. The microfluidic devices enabled the continuous fabrication of bioactive hollow microtubes with tunable dimensions. The novel hydrogels composed of alginate and fibrin have been developed and optimized in terms of mechanical strength, stability, biocompatibility and promotion of cell adhesion. The hydrogel microtubes can be prepared efficiently, rapidly and in large quantities. Compared to traditional migration experiments, the microtube model better simulated the *in vivo* environment in which the cells have normal morphology and characteristics. Through the microtubule model, all three NSAIDs have the anti-tumor migration ability, with nimesulide being the most effective, which provided a basis for further experiments. The microtube model has a bright future for large-scale clinical drug screening.

## Data Availability

The raw data supporting the conclusions of this article will be made available by the authors, without undue reservation.
