# Supplementary material for: Controlled Fabrication of Bioactive Microtubes for Screening Anti-Tongue Squamous Cell Migration Drugs
Source: Front Chem. 2022 Jan 21;10:771027. doi: 10.3389/fchem.2022.771027 (PMC8813861; doi:10.3389/fchem.2022.771027)
Supplement: Supplementary file 1 [file DataSheet3.docx]

**Preparation of microfluidic device times 24 h-48 h**

1. Design the two fixture parts and a PDMS master mold by the Autodesk Auto CAD 2014 and print them by a 3D Printer for 24 h.
2. Mix the curing agent and the precursor (1:10) of PDMS, and centrifuge for discharging the bubbles (4500 g, 5 min). Pour the mixed liquid into the PDMS master mold that we printed and solidified at room temperature for 12 h. A hard plastic tube with a diameter of 1.5mm was inserted in the master mold in advance.
3. Take out the plastic tube and remove the hard PDMS from the mold.

**Preparation of hydrogel microtubes model containing cells times 24 h-72 h**

1. Prepare 3% sodium alginate solution, 100 mM calcium chloride solution containing 3% sucrose, and 290 mM sodium chloride solution and perform high temperature sterilization for 3 h.
2. Weigh 50 mg of fibrinogen and dissolve it to 5 mg/ml in sterile saline and dissolve thrombin to 4 U with PBS.
3. Digest Cal27 cells by trypsin, count with a hemocytometer, incubate with 500 μL of 20 μM CFSE staining solution and 500 uL of PBS solution for 30 minutes, add 1mL of fetal bovine serum for 1 min, wash with PBS for 2 to 3 times, use a 1:1 mixture of sodium alginate solution and fibrinogen to adjust the number of cells to 1×10^6^.
4. Connect the whole microfluidic device, and slowly inject ethanol for 1 hour to sterilize, the PDMS is sterilized by UV irradiation for more than 1 h.
5. Fill the device with outer and inner solution, introducing saline in the channel of the inner instead of the 100 mM CaCl_2_ solution for 10 min.
6. Insert the 30 G injection needle vertically with a mixing solution containing 1.0×10^6^ Cal27 cells, fix it with PDMS and fixed module.
7. Replace the inner tube liquid of the coaxial needle with a 100 mM calcium chloride solution containing 2% sucrose, and set the coaxial needle injection speed to 500 μL/min, and the 30G needle injection speed to 350 μL/min.
8. Form the desired length of the tube, switch the CaCl_2_ flow to the saline stream again and stop the pumps, transfer the cell microtubes into 4 mL of thrombin, incubate at 37°C for 15 min, then transfer to a 6-well plate and add 2 mL complete medium.
9. Different concentrations of NSAIDs are added to each group of microtubes and incubate the microtubes at 37ºC, in a water-saturated 5% CO_2_ environment for 72 h.

**Verification of the effect of anti-migration drugs in the microtubule model**

1. Transfer the microtubes in the culture medium to 0.2% sucrose solution for 1.5 h.
2. Transfer the microtubes to a 1:1 mixture of sucrose and O.C.T. solution for 2 h.
3. Transfer the microtubes to a fresh O.C.T. solution for 4 h.
4. Replace the O.C.T. solution with another fresh O.C.T. solution for 6 hours.
5. The microtubes arrangement was adjusted to parallel, and frozen at -80°C for 30 min.
6. Separate and fix the microtubes on the holder of the freezing microtome and slice them into 20 μm sections, which were observed through the confocal laser scanning microscope.
