# Supplementary material for: Controlled Fabrication of Bioactive Microtubes for Screening Anti-Tongue Squamous Cell Migration Drugs
Source: Front Chem. 2022 Jan 21;10:771027. doi: 10.3389/fchem.2022.771027 (PMC8813861; doi:10.3389/fchem.2022.771027)
Supplement: Supplementary file 3 [file DataSheet1.docx]

Supplementary Material

## Supplementary Figures

**Supplementary Figure 1.**

(A) Preparation of hydrogel microtube device; (B) Silicon hose connects the coaxial needle inlet and outlet; (C) The image of hydrogel microtubes, with a diameter of 1.5 mm.

**
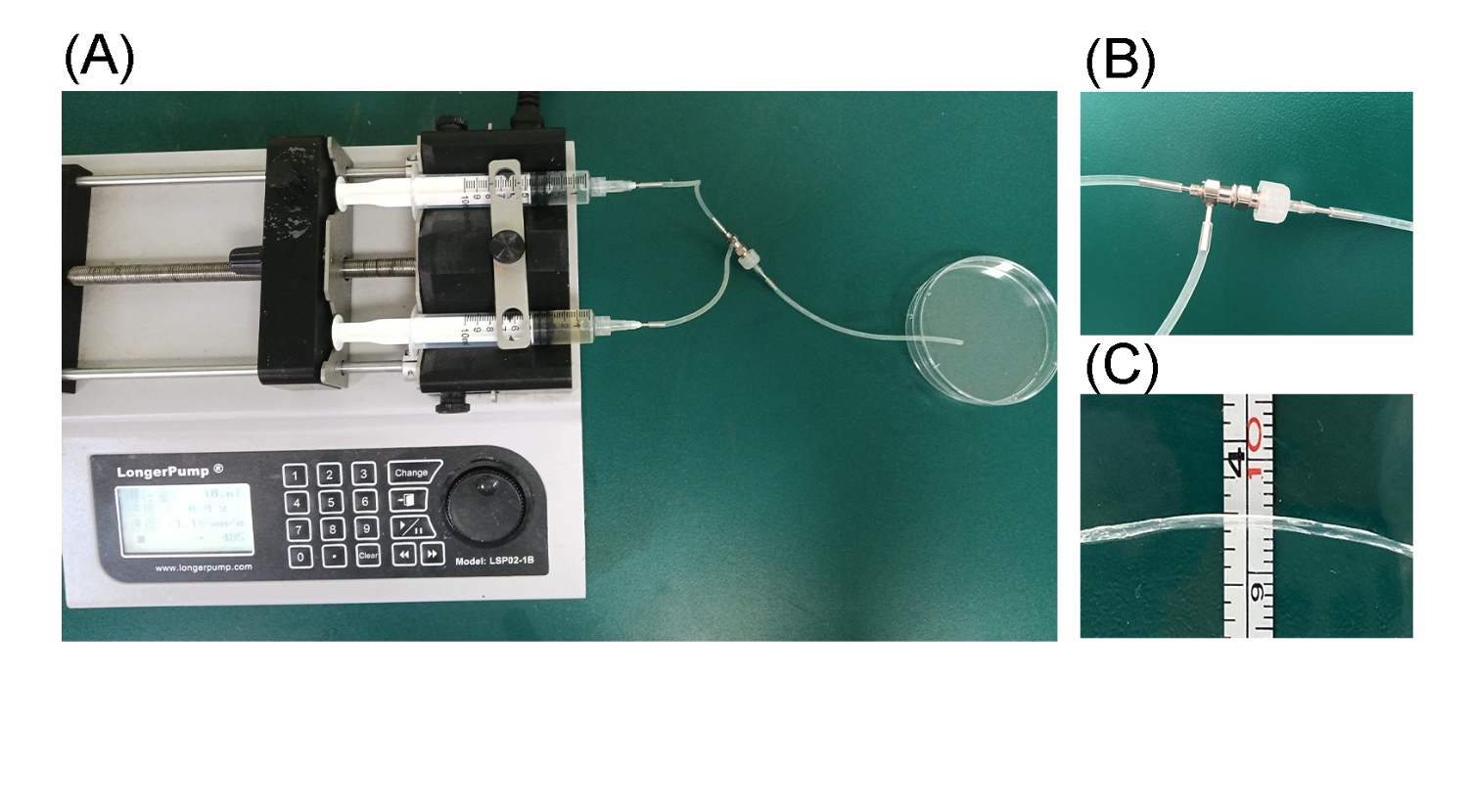
**

**Supplementary Figure 2.**

The influence of inner and outer liquid flow rate on microtube structure.

**
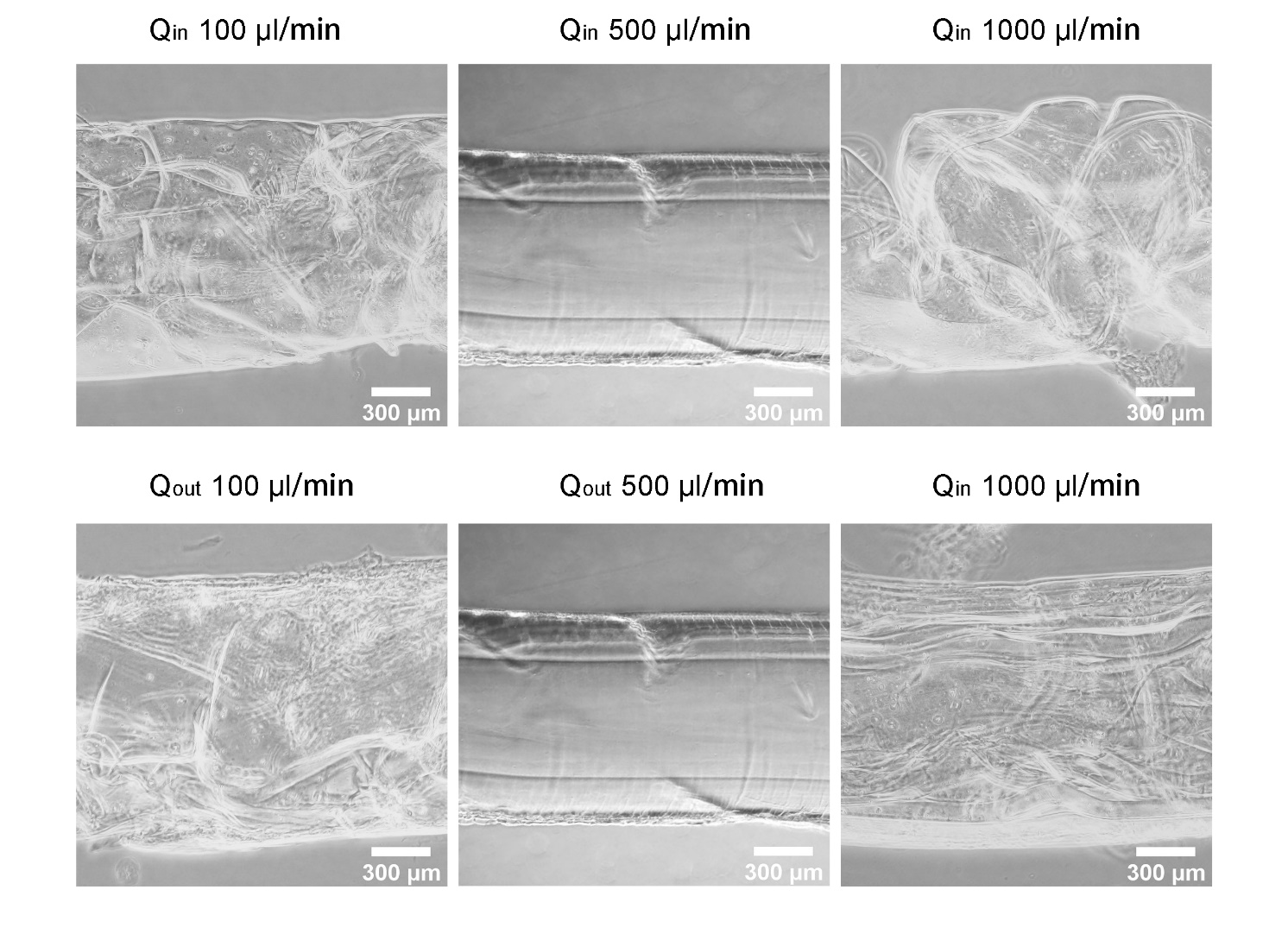
**

**Supplementary Figure 3.**

The effect of vertical injection depth on microtubule structure. (A) Microscopic image of the PDMS fixed block inserted a 30 G injection needle vertically, depth to the surface of microtubules, the scale is 800 μm; (B, C) Microscope marks the depth of injection perpendicular to the needle, the measuring depth is 115.664 μm, the scales are 500 μm and 200 μm, respectively; (D) Microscopic image of the PDMS fixed block inserted a 30 G injection needle vertically, depth to the center of the microtubule, the scale is 800 μm; (E, F) Microscope marks the depth of injection perpendicular to the needle, the measuring depth is 814.256 μm, the scales are 500 μm and 200 μm, respectively.

**
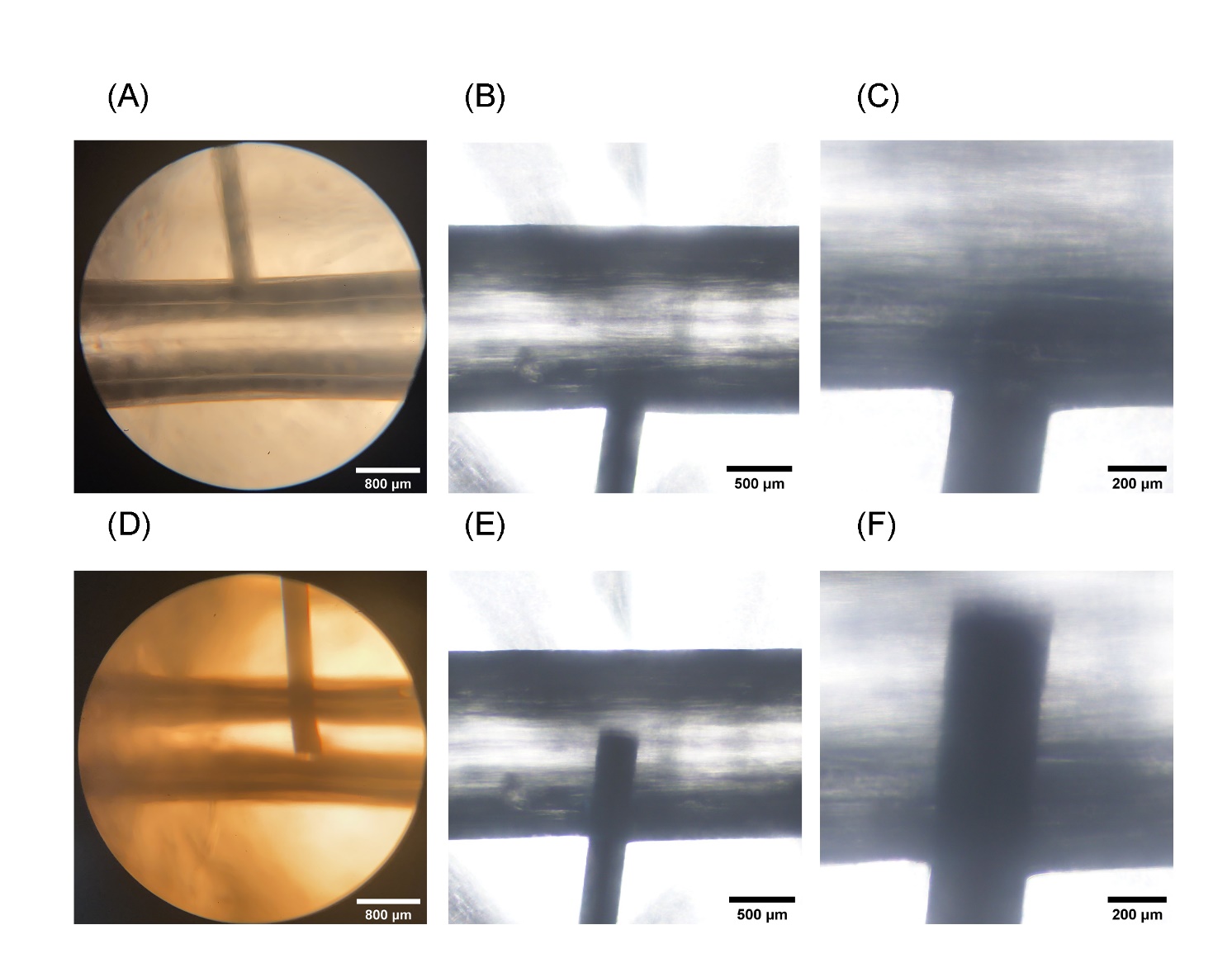
**

**Supplementary Figure 4.**

(A) Preparation of the mold design drawing of the PDMS fixture; (B) The image of the mold for preparing the PDMS fixture; (C) Design drawing of 3D printing fixture part 1; (D) The image of 3D printing fixture part 1; (E) Design drawing of 3D printing fixture part 2; (F) The image of 3D printing fixture part 2; (G) Install the 3D printing fixture to fix the coaxial needle.

**
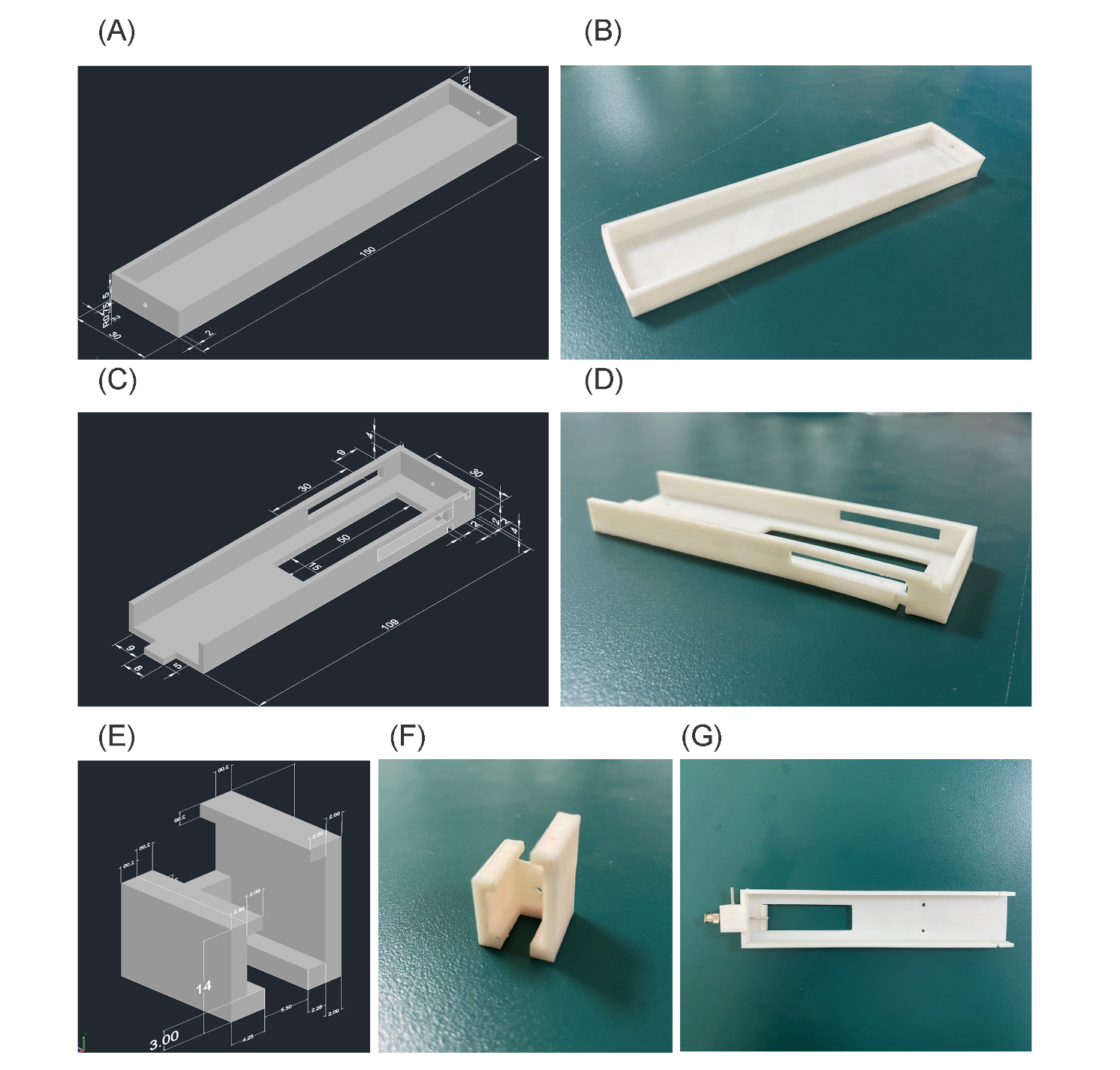
**

**Supplementary Figure 5.**

The picture of connecting the overall injection device


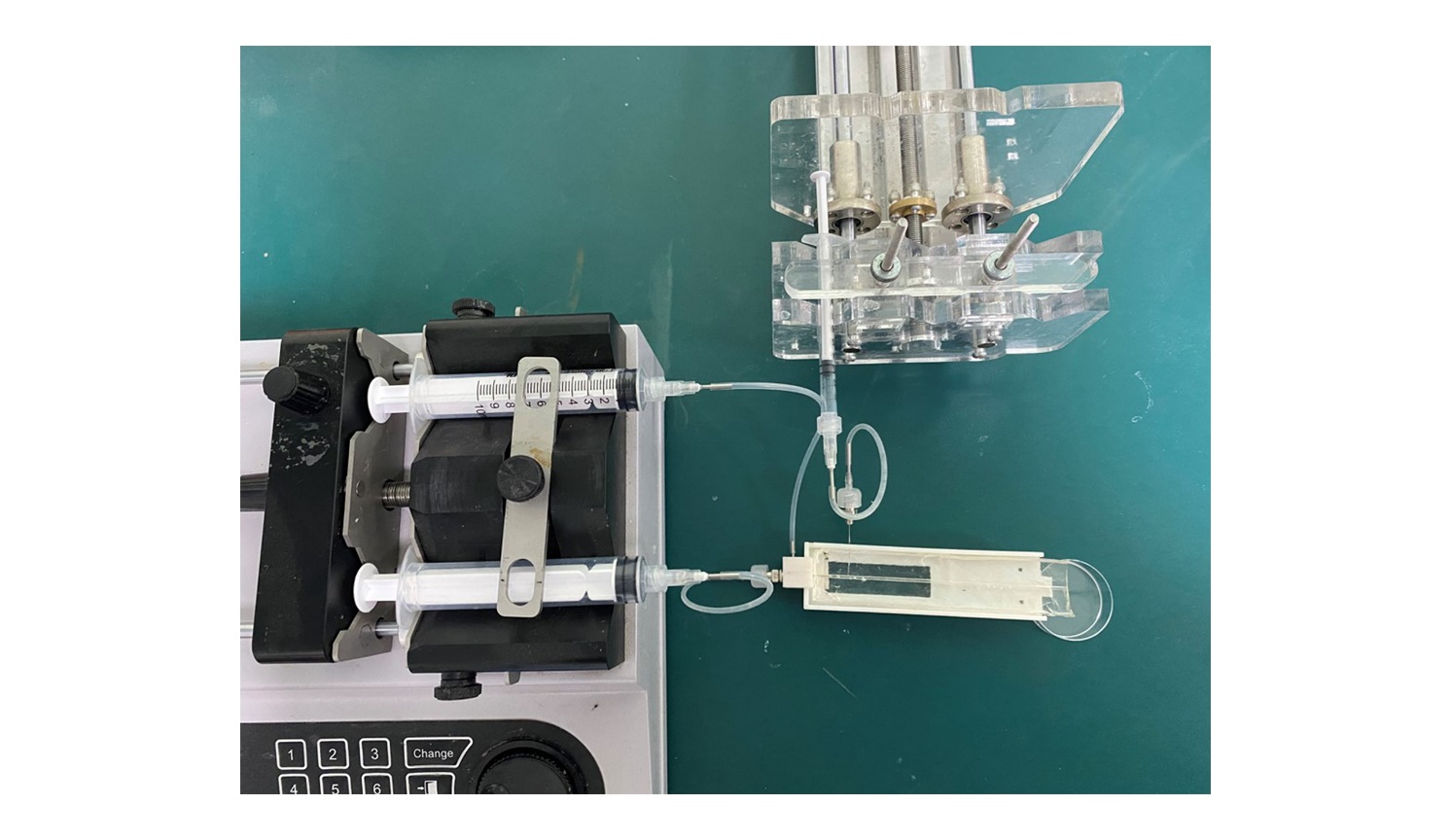


**Supplementary Figure 6.**

(A) Confocal microscopy images showing Cal27 on the surface of hydrogel with ECM for 24 h. The Cal27 cells were treated stained with TRITC-Pha (for actin staining), and observed by confocal microscopy. DAPI = 4,6-diamidino-2-phenylindole. The scale is 100 μm.

**
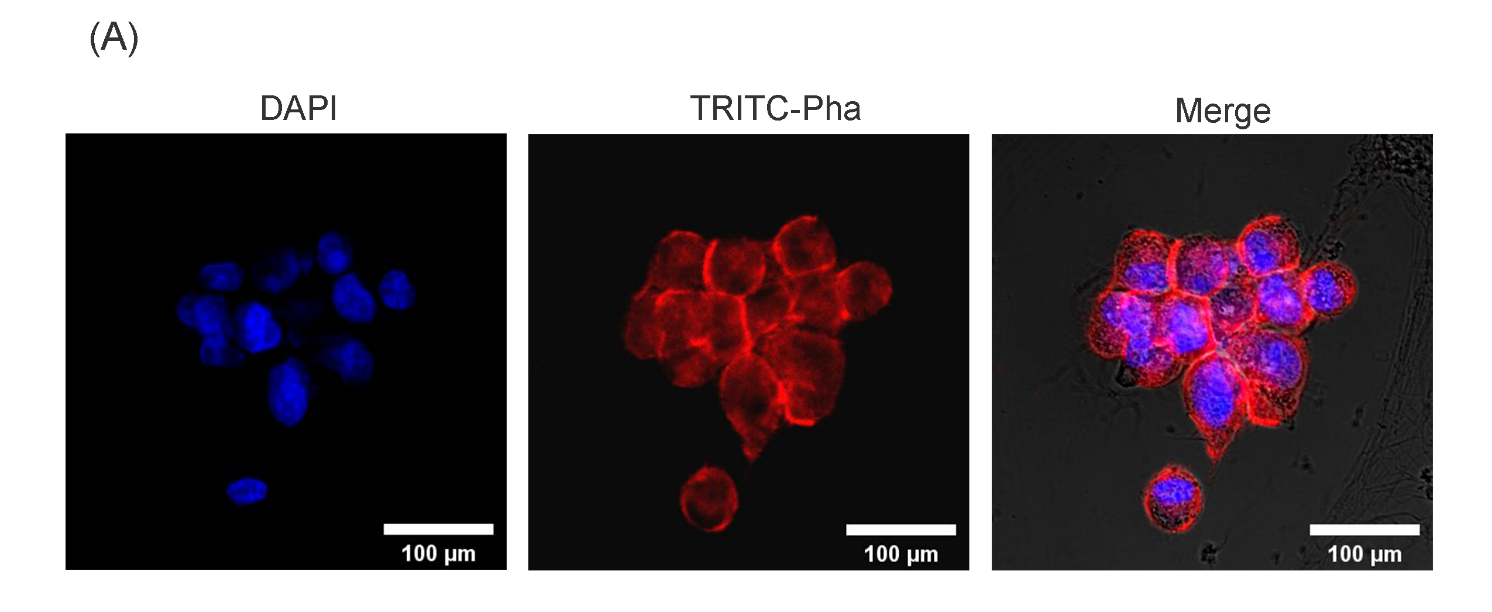
**

**Supplementary Figure 7.**

(A.C) Cells in the simple hydrogel group leaked from the microtubes, attached to the cell culture flask and grew for 96 h, with clear morphology, the scales were 500 μm and 200 μm, respectively; (B, D) Calcein-AM labeled live cells are green fluorescence, the scales are 500 μm and 200 μm, respectively.


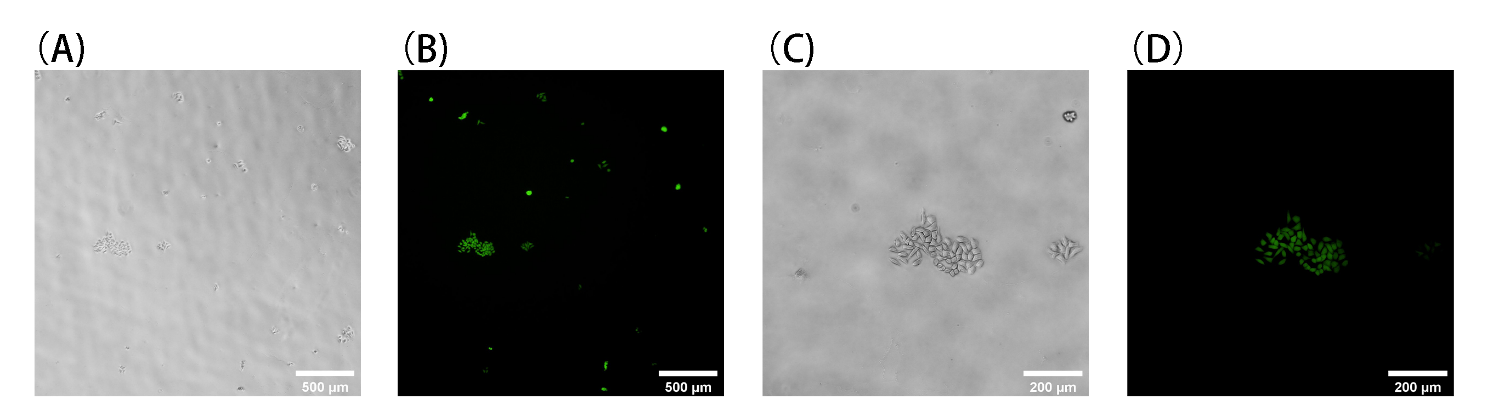


**
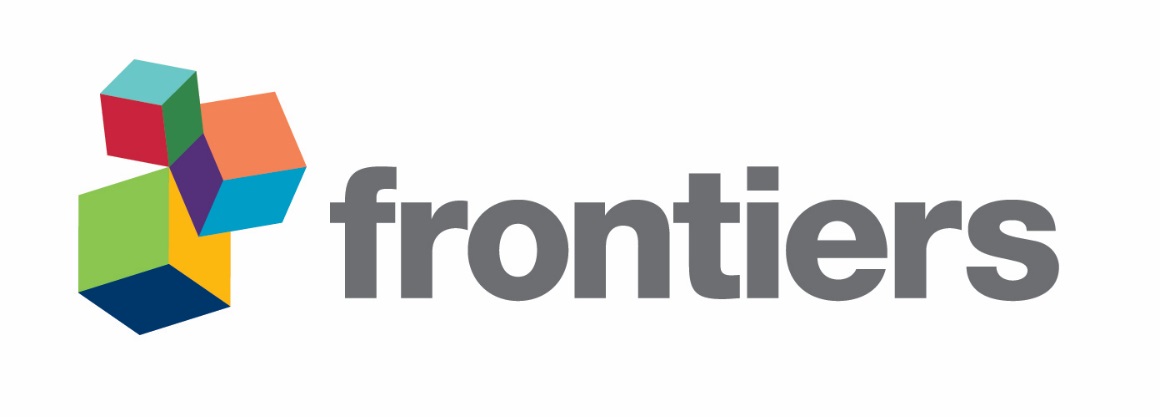
**
